# Heterogeneous network epidemics: real-time growth, variance and extinction of infection

**DOI:** 10.1007/s00285-016-1092-3

**Published:** 2017-01-17

**Authors:** Frank Ball, Thomas House

**Affiliations:** 1 School of Mathematical Sciences, University of Nottingham, University Park, Nottingham, NG7 2RD UK; 2 School of Mathematics, University of Manchester, Oxford Road, Manchester, M13 9PL UK

**Keywords:** SIR epidemic, Configuration model, Branching process, 92D30, 60J85, 05C80

## Abstract

Recent years have seen a large amount of interest in epidemics on networks as a way of representing the complex structure of contacts capable of spreading infections through the modern human population. The configuration model is a popular choice in theoretical studies since it combines the ability to specify the distribution of the number of contacts (degree) with analytical tractability. Here we consider the early real-time behaviour of the Markovian SIR epidemic model on a configuration model network using a multitype branching process. We find closed-form analytic expressions for the mean and variance of the number of infectious individuals as a function of time and the degree of the initially infected individual(s), and write down a system of differential equations for the probability of extinction by time *t* that are numerically fast compared to Monte Carlo simulation. We show that these quantities are all sensitive to the degree distribution—in particular we confirm that the mean prevalence of infection depends on the first two moments of the degree distribution and the variance in prevalence depends on the first three moments of the degree distribution. In contrast to most existing analytic approaches, the accuracy of these results does not depend on having a large number of infectious individuals, meaning that in the large population limit they would be asymptotically exact even for one initial infectious individual.

## Introduction

### Background

Models of infectious disease transmission have, from relatively modest beginnings (e.g. Bailey 1957), developed a rich domain of applicability covering the whole spectrum of human, animal and plant pathogens, and informing the study of questions from viral evolution, through epidemiology of infectious diseases, to public health policy (see Heesterbeek et al. 2015). Increasingly, networks have been seen as a way of modelling the complex, heterogeneous patterns of contacts between individuals (Danon et al. 2011).

In theoretical studies, the configuration model has been a popular choice due to the ability to specify the number of contacts each individual has that are capable of spreading disease, while allowing for analytic results to be obtained (e.g. Molloy and Reed 1995; Newman 2002). Ball and Neal (2008) used an effective degree approach (which we describe in Sect. 2.2 below—cf. Lindquist et al. 2010) to derive a system of ordinary differential equations that describes the deterministic limit of the epidemic model as the population size $N \rightarrow \infty$. A much simpler (equivalent) system of only 4 ordinary differential equations was obtained by Volz (2008) and subsequently shown by Miller (2011), Miller et al. (2012) to be essentially one-dimensional (the 4 ODEs were also shown by House and Keeling (2010) to be a special case of the much higher dimensional pair approximation model of Eames and Keeling (2002), in which the degree structure is explicit). Fully rigorous proofs of convergence in probability of the scaled stochastic model to the deterministic limit are given by Decreusefond et al. (2012), Bohman and Picollelli (2012), Barbour and Reinert (2013) and Janson et al. (2014). These works are primarily concerned with the temporal behaviour of *proportions* of the population in different epidemiological compartments (susceptible, infectious and removed) over the main body of a large epidemic. Here, we are also concerned with temporal behaviour, but focus on *numbers* infected early in the epidemic, including the possibility of early stochastic extinction.

In a recent paper, Graham and House (2014) use a pairwise approximation in conjunction with the central limit theorem for density dependent population processes (Ethier and Kurtz 1986, Chap. 11) to obtain a closed-form approximation to the mean and variance of prevalence in the linearised model which approximates the early asymptotic exponential growth phase of a Markovian SIR epidemic on a configuration network. In particular, they find that, under these approximations, the variance in disease prevalence is determined by the first three moments of the network degree distribution. In this paper, we use the effective degree approach of Ball and Neal (2008) to approximate the early stages of the epidemic by a continuous-time, multitype Markovian branching process, which is then analysed in detail. For $t\ge 0$, let *Z*(*t*) denote the total number of individuals alive in this branching process at time *t*, so *Z*(*t*) approximates disease prevalence in the epidemic model during its early asymptotic exponential growth phase. Explicit closed-form expressions are derived for the mean and variance of *Z*(*t*), the covariance of *Z*(*t*) and *Z*(*s*) to give the behaviour over time, and also for the probability of extinction $\pi (t) = \mathbb {P}(Z(t)=0)$. As in Graham and House (2014), the mean and variance in disease prevalence depends on the degree distribution only through its first two and three moments, respectively.

The results in Graham and House (2014) assume implicitly that the initial number of infectives is sufficiently large for the density dependent population process central limit theorem to yield a good approximation. In contrast, our results assume any arbitrary, but specified, initial number of infectives. The asymptotic distribution of types in the branching process, when it does not go extinct, is also available in closed-form and enables us to obtain a Gaussian process approximation, with explicit mean and covariance function, for the prevalence in the early asymptotic exponential growth phase of an SIR epidemic, with few initial infectives, which takes off and becomes established. We show that this approximation can be applied together with the methods of Ross et al. (2006) to estimate epidemiological parameters from early prevalence data of a simulated epidemic provided the first three moments of the degree distribution are known.

### Outline of the paper

The paper is organised as follows. The configuration network model and a Markov SIR epidemic on that network are described in Sect. 2.1. The effective degree construction of this epidemic is outlined in Sect. 2.2. Approximation of the early stages of this epidemic by a branching process is outlined in Sect. 2.3, where conditions are given for the mean, variance and covariance functions of the number of infectives in the epidemic process to converge to the corresponding quantities of the approximating branching process as the population size tend to infinity. The representation of the approximating branching process as a continuous-time, multitype Markov branching process is outlined in Sect. 2.3 and described more explicitly in Sect. 2.4. The mean, variance and covariance functions of the total number of individuals alive in the branching process are considered in Sects. 3, 4 and 5, respectively. Explicit closed-form expressions are obtained for each of these quantities and for their limits as time $t \rightarrow \infty$. The arguments in Sects. 3, 4 and 5 assume that underlying degree distribution has a maximum degree. In Sect. 6, we show that these expressions continue to hold in the unbounded degree setting, subject to the degree distribution satisfying suitable moment conditions. The probability that the branching process is extinct at time *t* is studied in Sect. 7. Closed-form expressions for this probability, given the initial state of the branching process, are not available so asymptotic results as $t \rightarrow 0$ and $t \rightarrow \infty$ are considered.

The mean, variance and covariance functions derived in Sects. 3, 4 and 5 are unconditional, so they include realisations of the branching process which result in extinction. However, in the epidemic setting, we are often interested in analysing the behaviour of epidemics that take off and become established, which correspond to non-extinction of the branching process. In Sect. 8, we first derive the mean and variance of the total number of individuals alive in the branching process at time *t*, conditional upon the process having survived to time *t*; fully closed-form results are not available owing to the absence of a closed-form expression for the survival probability. We then consider realisations of the branching process which reach some specified size, *K* say, with time being set to zero the first time the total number of individuals alive is *K*. The results in Sect. 3 yield an explicit expression for the asymptotic distribution of types, given that the branching process does not go extinct, which, provided *K* is sufficiently large, enables the above branching process starting from *K* individuals to be approximated by a Gaussian process whose mean and covariance functions are determined explicitly. The theory is illustrated by numerical examples of both forward simulation and inference in Sect. 9 and some concluding comments are given in Sect. 10.

In general, we define notation as it is introduced; we also collect notation that is used in multiple sections in Table 1.

**Table 1:** Notation used in multiple sections of this paper

Primary notation	Meaning	Equivalent notation
*Network properties*		
*N*	Size of the population	
*D*	A random variable for an individual’s degree	$D_1, D_2, \ldots$
$p_k$	Probability mass function for *D* evaluated at *k*	
$k_{\mathrm {max}}$	The maximum degree	
$\mathscr {K}$	The set of possible degrees	$\{0,1,\ldots k_{\mathrm {max}}\}$
${\tilde{D}}$	A random variable for an individual’s size-biased degree	${\tilde{D}}_1, {\tilde{D}}_2, \ldots$
${\tilde{p}}_k$	Probability mass function for ${\tilde{D}}$ evaluated at *k*	$\mu _D^{-1}kp_k$
*Vectors and matrices*		
$\mathbf {v}$	A column vector whose *k*th entry is $v_k$	$(v_k)$
$\mathbf {v} ^{\!\top }$	A row vector (transpose of a column vector)	
$\varvec{M}$	A matrix with (*k*, *l*)th entry $M_{kl}$ or $m_{kl}$	$[M_{kl}]$, $[m_{kl}]$
$| \varvec{M} |$	Determinant of matrix $\varvec{M}$	
$\mathbf {1}$	A column vector whose entries are all equal to 1	
$\mathbf {n}$	A column vector whose *i*th entry is *i*	
$\mathbf {n} _2$	A column vector whose *i*th entry is $i^2$	
$\varvec{I}$	The identity matrix	$[\delta _{k,l}]$
*Probability*		
$\mathbb {P}(e)$	Probability of event *e*	
$\mu _{f(X)}$	Expected value of a function *f* of a random variable *X*	$\mathbb {E}\left[ f(X)\right]$
$M_X(\theta )$	Moment generating function for random variable *X*	$\mathbb {E}\left[ \mathrm {exp}(\theta X)\right]$
$\mathrm {var}(X)$	Variance of random variable *X*	$\mathbb {E}\left[ X^2\right] - \mathbb {E}\left[ X\right] ^2$
$\mathrm {cov}(X,Y)$	Covariance of random variables *X* and *Y*	$\mathbb {E}\left[ X Y\right] - \mathbb {E}\left[ X\right] \mathbb {E}\left[ Y\right]$
*Epidemic and branching process dynamics*		
$\tau$	Rate of transmission across a network link	
$\gamma$	Rate of recovery from infection	
$\omega _k$	Death rate for individual of type *k*	
*t*	Real time	*s*
$\mathscr {B}$	The limiting branching process	
$E_N$	The epidemic process in a population of size *N*	
*K*	A large value of infectious population size	
$Z^{(k)}_i(t)$	Random number of individuals of type *i* in the branching process at time *t* given initial type *k*	
$\pi _k(t)$	Probability that the branching process is extinct at time *t* given initial type *k*	$1-q_k(t)$

## Model and approximating branching process

### Model

We consider the spread of an SIR epidemic on a network of *N* individuals, labelled $1,2,\ldots ,N$, constructed using the configuration model as follows (see e.g. Newman 2002). Let *D* be a random variable which describes the degree of a typical individual and let $p_k=\mathbb {P}(D=k)$ $(k=0,1,\ldots )$. Let $D_1,D_2,\ldots ,D_N$ be independent realisations of *D* and, for $i=1,2,\ldots , N$, attach $D_i$ stubs (half-edges) to individual *i*. Pair up these stubs uniformly at random to form the edges in the network. If $D_1+D_2+\cdots +D_N$ is odd, there will be a left-over stub, which is ignored; the resulting network may have other ‘defects’ such as self-loops and multiple edges between pairs of individuals but, provided that *D* has finite variance, such imperfections become sparse in the network as $N \rightarrow \infty$ (see e.g. Durrett 2007, Theorem 3.1.2). An alternative to the degrees $D_1,D_2,\ldots ,D_N$ being random is, for each $N=1,2,\cdots$, to replace $\mathbf {D} =(D_1,D_2,\ldots ,D_N)$ by $\mathbf {D} ^{(N)}=\left( D_1^{(N)},D_2^{(N)},\ldots ,D_N^{(N)}\right)$, where the degree sequences $\mathbf {D} ^{(N)}$ $(N=1,2,\ldots )$ are prescribed and satisfy $p_k^{(N)}=N^{-1} \sum _{i=1}^N \delta _{k,D_i^{(N)}} \rightarrow p_k$ as $N \rightarrow \infty$ $(k=0,1,\ldots )$, where the Kronecker delta $\delta _{k,j}$ is 1 if $k=j$ and 0 otherwise (see e.g. Molloy and Reed 1995).

The epidemic is defined as follows. Initially some individuals are infective and the remaining individuals are susceptible. Infective individuals have independent infectious periods, each having an exponential distribution with rate $\gamma$ (and hence mean $\gamma ^{-1}$), after which they become recovered and play no further role in the epidemic. Throughout its infectious period, an infective contacts each of its susceptible neighbours in the network at the points of independent Poisson processes having rate $\tau$, so the probability that a given infective contacts a given neighbour before the infective recovers is $\tau /(\gamma +\tau )$. Any contacted susceptible immediately becomes infective and may transmit the infection to any of its neighbouring susceptibles; i.e. there is no latent period. All the infectious periods and Poisson processes governing transmission of infection are mutually independent. The epidemic ends as soon as there is no infective present in the network.

### The effective degree model

Ball and Neal (2008) introduced an ‘effective-degree’ construction of the above epidemic, in which the network is constructed as the epidemic progresses. The process starts with some individuals infective and the remaining individuals susceptible, but with none of the stubs paired up. For $i=1,2,\ldots ,N$, the effective degree of individual *i* is initially $D_i$. Infected individuals transmit infection by pairing their stubs with stubs attached to susceptible individuals in the following fashion. An infected individual makes infectious contacts down its unpaired stubs independently at rate $\tau$ and is removed at rate $\gamma$. When an infective, individual *i* say, transmits infection down a stub that stub is paired with a stub (attached to individual *j*, say) chosen independently and uniformly at random from all the unpaired stubs, to form an edge. The effective degrees of individuals *i* and *j* are both reduced by 1. If $i=j$ then the effective degree of individual *i* is reduced by 2 but this will not significantly affect the dynamics for large populations since the probability of it happening is $O(N^{-1}$). If individual *j* is susceptible then it becomes infective and can transmit infection down any of its unattached stubs. As before, the epidemic ends as soon as there is no infective present. The network is then typically only partially constructed but that does not matter if interest is focussed on properties of the epidemic. In the original formulation of Ball and Neal (2008), when an infective recovers its unpaired stubs, if any, were paired with stubs chosen uniformly at random without replacement from the set of unpaired stubs but that is unnecessary; the stubs from such an infective can simply be left in the set of unpaired stubs.

### Approximating multitype branching process

Suppose that the size *N* of the network is large and the initial number of infectives is small. Then during the early stages of an epidemic it is very likely that each time an infective individual transmits infection down a stub that stub is paired with a stub belonging to a susceptible individual. It follows that the early stages of such an epidemic can be approximated by a branching process in which each newly-infected individual has their “full” effective degree (i.e. their actual degree minus one for the stub that is paired with their infector). This approximation can be made fully rigorous by considering a sequence of epidemics, indexed by *N*, and using a coupling argument; see e.g. Ball and Neal (2008), which treats a more general model in which infective individuals also make contacts with individuals chosen uniformly at random from the population. Let $E_N$ denote the epidemic on a network of *N* individuals and let $\mathscr {B}$ denote the approximating branching process. Then following Ball and Neal (2008) (see “Appendix 1”) if $\mu _D=\mathbb {E}\left[ D\right]$ is finite then the epidemics $E_1,E_2\dots$ and the branching process $\mathscr {B}$ can be constructed on a common probability space so that, with probability one, over any finite time interval [0, *t*] the process of infectives in $E_N$ and the branching process $\mathscr {B}$ coincide for all sufficiently large *N*. The same result holds for the model with prescribed degree sequences provided that $p^{(N)}_k \rightarrow p_k$ $(k=0,1,\ldots )$ and $\mu _D^{(N)}=\sum _{k=0}^\infty p^{(N)}_k k \rightarrow \mu _D$ as $N \rightarrow \infty$, where $\sum _{k=0}^{\infty } p_k=1$ and $\mu _D < \infty$.

As indicated in “Appendix 1”, the branching process $\mathscr {B}$ is not an almost sure upperbound for the process of infectives in $E_N$, so unlike in Theorem 3.1 of Ball and Donnelly (1995) which considers homogeneously mixing epidemics, one cannot simply use the dominated convergence theorem to deduce convergence of moments of the number of infectives in the epidemic process to corresponding moments of the branching process as $N \rightarrow \infty$. If there is a maximum degree $k_{\mathrm {max}}$ (i.e. $p_k=0$ for all $k>k_{\mathrm {max}}$, or $p_k^{(N)}=0$ for all $k>k_{\mathrm {max}}$ and all *N* in the model with prescribed degrees) then, for all *N*, the process of infectives in $E_N$ is bounded above by a branching process in which each newly-infected individual has the maximun effective degree $k_{\mathrm {max}}-1$, so in that case the dominated convergence theorem can be used to prove convergence of moments. In “Appendix 1”, we consider the case when there is no maximum degree and use uniform integrability arguments to determine sufficient conditions for the mean and variance of the number of infectives at any given time $t \ge 0$, and the covariance of the number of infectives at any given times $t,s \ge 0$, in the epidemic $E_N$ to converge to the corresponding mean, variance and covariance of the branching process $\mathscr {B}$ as $N \rightarrow \infty$. Specifically, we prove that (i) in the model with prescribed degrees these moments converge if, in addition to the conditions given above, there exists $\delta >0$ such that $\mu _{D^{3+\delta }}^{(N)}=\sum _{k=0}^\infty p^{(N)}_k k^{3+\delta } \rightarrow \mu _{D^{3+\delta }}=\sum _{k=0}^\infty p_k k^{3+\delta }$ as $N \rightarrow \infty$, where $\mu _{D^{3+\delta }} < \infty$; and (ii) in the model with random degrees they converge if the moment-generating function $M_{D^2}(\theta )=\mathbb {E}\left[ \exp \left( \theta D^2\right) \right]$ of $D^2$ is finite for some $\theta _0>0$. Note that the latter condition implies that $\mathbb {E}\left[ D^\alpha \right] < \infty$ for all $\alpha \ge 0$.

In this context, we note that, for the model with prescribed degrees, the weakest conditions obtained on the moments of the degree distribution for convergence of the scaled stochastic epidemic on to its deterministic limit are given by Janson et al. (2014), who require uniform boundedness of the second moment of $\mathbf {D} ^{(N)}$. However that paper, and the other related papers cited in the second paragraph of Sect. 1.1, (i) are concerned with the entire time course of the epidemic; (ii) assume that either the epidemic starts with a positive fraction of the population infected in the limit as $N \rightarrow \infty$, or if that limiting fraction is zero then the convergence is for epidemics which take off and involves a random time translation describing when the epidemic becomes suitably established; and (iii) consider the evolution of the proportion of the population that is susceptible, infective or recovered. By contrast, this paper is concerned with epidemics initiated by few infectives and considers the number, rather than proportion, of infectives during the early phase of such an epidemic. Under the coupling mentioned above, in the limit as $N \rightarrow \infty$, if an epidemic takes off then the duration of its early (exponentially growing) phase tends almost surely to infinity.

The limiting branching process may be described by a continuous-time multitype Markov branching process, with the type of an infective corresponding to its effective degree. Let ${\tilde{D}}$ denote the (size-biased) degree of a typical neighbour of a typical individual in the network and let ${\tilde{p}}_k=\mathbb {P}({\tilde{D}}=k)$ $(k=1,2,\ldots )$. Then ${\tilde{p}}_k=\mu _D^{-1} k p_k$, where $\mu _D=\mathbb {E}\left[ D\right]$, since when a stub is paired it is *k* times as likely to be paired with a stub from a given individual having degree *k* than it is with a stub from a given individual having degree 1. Under the branching process approximation, the effective degree of a newly infected individual is distributed according to ${\tilde{D}}-1$, since one of that individual’s stubs is ‘used up’ when it is infected. Note for future reference that $\mu _{{\tilde{D}}}=\mu _D^{-1}\mu _{D^2}$ and, more generally, $\mu _{f({\tilde{D}})}=\mu _D^{-1} \mu _{Df(D)}$ for any real-valued function *f*. (For a random variable, *X* say, we use $\mu _X$ to denote its expectation $\mathbb {E}\left[ X\right]$. Thus, for example, $\mu _{Df(D)}=\mathbb {E}\left[ Df(D)\right]$.)

### Explicit form for the multitype branching process

We now assume that there is a maximum degree $k_{\mathrm {max}}$. We show in Sect. 6 that our results for moments of the branching process extend to the case of no maximum degree size, subject to suitable moment conditions on *D*. Thus the type space for the branching process is $\mathscr {K}=\{0,1,\ldots ,k_{\mathrm {max}}\}$. Note that only initial infectives can have type $k_{\mathrm {max}}$. For $k\in \mathscr {K}$, an individual of type *k* dies if either its infectious period comes to an end or it transmits infection down one of its unattached stubs, whichever happens first. If the former happens first then the individual has no offspring, otherwise it has two offspring, namely an individual of type $k-1$ and an individual whose type is distributed according to ${\tilde{D}}-1$. Note that an individual of type 0 necessarily has no offspring when it dies. Thus, for $k\in \mathscr {K}$, the lifetime of an individual of type *k* has an exponential distribution with rate

2.1 $$\begin{aligned} \omega _k = \gamma + \tau k, \end{aligned}$$

and when it dies its offspring is distributed as follows (recall that ${\tilde{p}}_{k_{\mathrm {max}}+1}=0$):

2.2 $$\begin{aligned} \mathbb {P}\left( \mathrm {Offspring} = \varnothing | \mathrm {Parent\ type} = k \right)&= \frac{\gamma }{\gamma + \tau k}, \nonumber \\ \mathbb {P}\left( \mathrm {Offspring} = \{k-1,l\} | \mathrm {Parent\ type} = k \right)&= \frac{\tau k {\tilde{p}}_{l+1}}{\gamma + \tau k} \quad (l=0,1,\ldots ,k_{\mathrm {max}}-1) . \end{aligned}$$

The joint probability-generating function (PGF) for offspring of a type-*k* individual is therefore

2.3 $$\begin{aligned} P_k( \mathbf {s} ) = \frac{1}{\omega _k} \left( \gamma + \tau k s_{k-1} \sum _{l=0}^{k_{\mathrm {max}}-1} {\tilde{p}}_{l+1} s_l \right) , \end{aligned}$$

where $\mathbf {s} =(s_k)$. In general we will write $\mathbf {v} = (v_k) = (v_0,v_1,\ldots ,v_{k_{\mathrm {max}}})^{\!\top }$ for a column vector in $\mathbb {R}^{k_{\mathrm {max}}+1}$, where $^\top$ denotes transpose. Verbally, we will call $v_0$ the 0th element of such a vector, $v_1$ the first element etc. For $k\in \mathscr {K}$, let $\varvec{\partial } P_k( \mathbf {s} )$ be the column vector whose *i*th element is $\frac{\partial P_k( \mathbf {s} )}{\partial s_i}$ and let $\varvec{\partial } ^2 P_{k}( \mathbf {s} )$ be the matrix whose (*i*, *j*)th element is $\frac{{\partial }^2 P_{k}( \mathbf {s} )}{\partial s_i \partial s_j}$. We note for future reference that, for $i,j,k\in \mathscr {K}$,

2.4 $$\begin{aligned} \left( \varvec{\partial } P_k(\mathbf {1})\right) _i&=\frac{\tau k}{\gamma +\tau k}\left( {\tilde{p}}_{i+1}+\delta _{k-1,i}\right) , \nonumber \\ \left[ \varvec{\partial } ^2 P_{k}(\mathbf {1})\right] _{i,j}&=\frac{\tau k}{\gamma +\tau k}\left( {\tilde{p}}_{i+1}\delta _{k-1,j}+{\tilde{p}}_{j+1}\delta _{k-1,i}\right) , \end{aligned}$$

where $\mathbf {1}$ is the length-$(k_{\mathrm {max}}+1)$ column vector of ones.

For $t \ge 0$, let $\mathbf {Z}(t)=\left( Z_i(t)\right)$, where $Z_i(t)$ denotes the number of individuals of type *i* alive at time *t*, and let $Z(t)=Z_0(t)+Z_1(t)+\cdots +Z_{k_{\mathrm {max}}}(t)= \mathbf {1}^{\!\top }\mathbf {Z}(t)$ denote the total number individuals alive at time *t*. For $k\in \mathscr {K}$, we use the notation $\{\mathbf {Z}^{(k)}(t):t \ge 0\}$, where $\mathbf {Z}^{(k)}(t)=(Z^{(k)}_i(t))$, to denote a process starting with a single individual, whose type is *k*, at time 0 (i.e. where $Z_i^{(k)}(0) = \delta _{i,k}$, $i \in \mathscr {K}$). Further, $Z^{(k)}(t)=\mathbf {1}^{\!\top }{\mathbf {Z}^{(k)}(t)}$ denotes the total number of individuals at time *t* in such a process.

## Behaviour of means

In the next three sections we consider the behaviour of the mean, variance and covariance function of the total number of individuals over time in the branching process which approximates the initial phase of an epidemic. For $t \ge 0$ and $i,j,k\in \mathscr {K}$, let

$$\begin{aligned} \varvec{M} (t)&=[m_{i,j}(t)], \text {where}\quad m_{i,j}(t)=\mathbb {E}\left[ Z^{(i)}_j(t)\right] , \\ \mathbf {m} ^{(k)}(t)&=\mathbb {E}\left[ \mathbf {Z}^{(k)}(t)\right] = \varvec{M} (t)^{\!\top }{ \mathbf {u} _k} , \\ m^{(k)}(t)&=\mathbb {E}\left[ Z^{(k)}(t)\right] =\mathbf {1}^{\!\top } \mathbf {m} ^{(k)}(t) = \mathbf {u} _k^{\!\top } \varvec{M} (t) \mathbf {1} , \end{aligned}$$

where $\mathbf {u} _k$ is a length-$(k_{\mathrm {max}}+1)$ column vector with *k*th element equal to 1 and other elements equal to 0. A standard argument using the Kolmogorov forward equation (see e.g. Dorman et al. 2004, Sect. 7 and recall that ${\tilde{p}}_{k_{\mathrm {max}}+1}=0$) then yields that

3.1 $$\begin{aligned} \frac{\mathrm {d}}{\mathrm {d}t} \varvec{M} (t)= \varvec{M} (t) \varvec{{\varOmega }} ,\qquad \varvec{M} (0)= \varvec{I} , \end{aligned}$$

where $\varvec{I}$ denotes the $(k_{\mathrm {max}}+1) \times (k_{\mathrm {max}}+1)$ identity matrix and $\varvec{{\varOmega }} =[{\varOmega }_{l,k}]$ is the $(k_{\mathrm {max}}+1) \times (k_{\mathrm {max}}+1)$ matrix with elements given by

$$\begin{aligned} {\varOmega }_{l,k} = \tau l \left( {\tilde{p}}_{k+1} + \delta _{l,k+1}\right) -(\gamma + \tau l) \delta _{l,k} \qquad (l,k \in \mathscr {K}) . \end{aligned}$$

The solution to (3.1) is then straightforwardly given by

3.2 $$\begin{aligned} \varvec{M} (t)=\mathrm{e}^{ \varvec{{\varOmega }} t} =\sum _{l=0}^\infty \frac{t^l \varvec{{\varOmega }} ^l}{l!} . \end{aligned}$$

We show in “Appendix 2” that the eigenvalues of $\varvec{{\varOmega }}$ are

3.3 $$\begin{aligned} \lambda _i = {\left\{ \begin{array}{ll} - \gamma - i \tau &{} \text { for } i \in \{0,2,3,\ldots ,k_{\mathrm {max}}\} ,\\ \tau \left( \left( {\textstyle \sum _{l=0}^{k_{\mathrm {max}}}} l {\tilde{p}}_{l+1}\right) -1\right) - \gamma &{} \text { for } i = 1 . \end{array}\right. } \end{aligned}$$

We denote the dominant eigenvalue, $\lambda _1$, by *r*, so

3.4 $$\begin{aligned} r= \tau \left( \left( {\textstyle \sum _{l=0}^{k_{\mathrm {max}}}} l {\tilde{p}}_{l+1}\right) -1\right) - \gamma = \tau \mu _{{\tilde{D}}-2} - \gamma . \end{aligned}$$

If $r \le 0$, the branching process $\{\mathbf {Z}(t):t \ge 0\}$ goes extinct almost surely. If $r>0$, then *r* gives the asymptotic exponential growth rate of $\{Z(t):t \ge 0\}$ (and also of $\{Z_i(t):t \ge 0\}$ for $i \in \mathscr {K} \setminus \{k_{\mathrm {max}}\}$) when $\{\mathbf {Z}(t):t \ge 0\}$ does not go extinct.

For $i\in \mathscr {K}$, let $\mathbf {w} _i^{\!\top } = (w_{i,k})$ be a left eigenvector of $\varvec{{\varOmega }}$ corresponding to the eigenvalue $\lambda _i$, so

3.5 $$\begin{aligned} \mathbf {w} _i^{\!\top } \varvec{{\varOmega }} = \lambda _i \mathbf {w} _i^{\!\top } . \end{aligned}$$

The Perron–Frobenius theory implies that $\mathbf {w} _1$ can be chosen so that all of its elements are positive and $\mathbf {w} _1^{\!\top } \mathbf {1}=1$. The left-eigenvector $\mathbf {w} _1$ then yields a probability distribution which gives the asymptotic relative frequencies of the different types, as $t \rightarrow \infty$, when $\{\mathbf {Z}(t):t \ge 0\}$ does not go extinct.

Expanding (3.5) in components yields

3.6 $$\begin{aligned} \sum _{l=0}^{k_{\mathrm {max}}} w_{1,l} \left( \tau l \left( {\tilde{p}}_{k+1} + \delta _{l,k+1}\right) -(\gamma + \tau l) \delta _{l,k} \right) = r w_{1,k} \quad (k\in \mathscr {K}) . \end{aligned}$$

Let $w(s)=\sum _{l=0}^{k_{\mathrm {max}}} s^l w_{1,l}$ $(s \ge 0)$ denote the (probability-)generating function of $\mathbf {w} _1$. Multiplying (3.6) by $s^k$ and summing over *k* yields

3.7 $$\begin{aligned} \tau f_{{\tilde{D}}-1}(s)\mu _W+\tau (1-s) w'(s)=(r+\gamma )w(s), \end{aligned}$$

where $f_{{\tilde{D}}-1}(s)=\sum _{k=1}^{k_{\mathrm {max}}} {\tilde{p}}_k s^{k-1}$ is the PGF of ${\tilde{D}}-1$ and $\mu _W=\sum _{k=0}^{k_{\mathrm {max}}} k w_{1,k}$ is the mean of the distribution $\mathbf {w} _1$. Setting $s=1$ in (3.7) and using (3.4) yields

3.8 $$\begin{aligned} \mu _W=\frac{r+\gamma }{\tau }=\mu _{{\tilde{D}}-2}. \end{aligned}$$

Note that (3.8) has a simple intuitive explanation. For large *t*, a typical individual gives birth at rate $\sum _{l=0}^{k_{\mathrm {max}}}w_{1,l} l \tau =\tau \mu _W$ and dies completely (i.e. without producing any offspring) at rate $\gamma$, so the population growth rate $r=\tau \mu _W-\gamma$ and (3.8) follows using (3.4) .

For $i,k \in \mathbb {Z}^+$, let $k_{[i]}=k(k-1)\ldots (k-i+1)$ denote a falling factorial, with the convention $k_{[0]}=1$. For $i=0,1,\ldots$, let $\mu _W^{[i]}=\sum _{k=0}^{k_{\mathrm {max}}} k_{[i]} w_{1,k}$ be the *i*th factorial-moment of the distribution $\mathbf {w} _1$, so $\mu _W^{[0]}=1$ and $\mu _W^{[1]}=\mu _W$. Note that $\mu _W^{[i]}=w^{(i)}(1)$ $(i=0,1,\ldots )$, where $w^{(i)}(s)$ denotes the *i*th derivative of *w*(*s*). Repeated differentiation of (3.7) yields

3.9 $$\begin{aligned} \mu _W^{[i]}=\frac{\mu _{{\tilde{D}}-2}}{\mu _{{\tilde{D}}-2+i}}\mu _{{\tilde{D}}-1}^{[i]}, \end{aligned}$$

where $\mu _{{\tilde{D}}-1}^{[i]}$ is the *i*th factorial-moment of ${\tilde{D}}-1$. Note that $\mu _{{\tilde{D}}-1}^{[i]}=0$ for $i \ge k_{\mathrm {max}}$. It then follows, using the inversion formula which expresses the probability mass function of a non-negative integer-valued random variable in terms of its factorial-moments (see e.g. Daley and Vere-Jones 1988, p. 117), that

3.10 $$\begin{aligned} w_{1,k}={\left\{ \begin{array}{ll} \sum _{i=k}^{k_{\mathrm {max}}-1} (-1)^{i-k} {i \atopwithdelims ()k}\frac{\mu _{{\tilde{D}}-2} \mu _{{\tilde{D}}-1}^{[i]}}{i! \mu _{{\tilde{D}}-2+i}} &{} \text { if } k=0,1,\ldots ,k_{\mathrm {max}}-1 ,\\ 0 &{} \text { if } k = k_{\mathrm {max}}. \end{array}\right. } \end{aligned}$$

Observe that $w_{1,k_{\mathrm {max}}}=0$ since only initial infectives can have type $k_{\mathrm {max}}$. Observe also that $\mathbf {w} _1$ is determined just by the degree distribution of the network and is invariant to the epidemic parameters $\gamma$ and $\tau$.

For $t\ge 0$, let $\mathbf {m} (t)=(m^{(i)}(t))=( \varvec{M} (t)\mathbf {1})^{\!\top }$. Thus the *k*th element of $\mathbf {m} (t)$ contains the mean total population size at time *t* given that the process starts with a single individual whose type is *k*. We derive a simple expression for $\mathbf {m} (t)$. The following proposition is useful.

### Proposition 1

For a matrix $\varvec{M}$ and vectors $\mathbf {x}$, $\mathbf {y}$ such that $\varvec{M} \mathbf {x} = a \mathbf {x} + b \mathbf {y}$ and $\varvec{M} \mathbf {y} = c \mathbf {y}$, where *a*, *b* and *c* are scalars satisfying $a \ne c$,

3.11 $$\begin{aligned} \mathrm{e}^{ \varvec{M} t} \mathbf {x} = \mathrm{e}^{at} \mathbf {x} + \frac{b}{a-c} \left( \mathrm{e}^{at} - \mathrm{e}^{ct}\right) \mathbf {y} \qquad \hbox {and}\qquad \mathrm{e}^{ \varvec{M} t} \mathbf {y} = \mathrm{e}^{c t} \mathbf {y} . \end{aligned}$$

### Proof

The second identity follows straightforwardly from the definition of the matrix exponential and the fact that $\mathbf {y}$ is a right eigenvector with eigenvalue *c*. For the first identity,

3.12 $$\begin{aligned} \mathrm{e}^{ \varvec{M} t} \mathbf {x}&= \sum _{i=0}^{\infty } \frac{1}{i!}( \varvec{M} t)^i \mathbf {x} \nonumber \\&= \sum _{i=0}^{\infty } \frac{t^i}{i!} \left( a^i \mathbf {x} + \sum _{j=0}^{i-1} b c^{i-1-j}a^j \mathbf {y} \right) \nonumber \\&= \mathrm{e}^{at} \mathbf {x} + \sum _{i=0}^{\infty } \frac{t^i}{i!} b c^{i-1} \frac{\left( \frac{a}{c}\right) ^i -1}{\left( \frac{a}{c}\right) -1} \mathbf {y} \nonumber \\&= \mathrm{e}^{at} \mathbf {x} + \frac{b}{a-c} \left( \mathrm{e}^{at} - \mathrm{e}^{ct}\right) \mathbf {y} . \end{aligned}$$

$$\square $$

Let $\mathbf {n} =(0,1,\ldots ,k_{\mathrm {max}})^{\!\top }$. Observe that

3.13 $$\begin{aligned} \varvec{{\varOmega }} \mathbf {1}= \tau \mathbf {n} - \gamma \mathbf {1}\qquad \text{ and }\qquad \varvec{{\varOmega }} \mathbf {n} = r \mathbf {n} , \end{aligned}$$

so using Proposition 1 with $\varvec{M} = \varvec{{\varOmega }} , \mathbf {x} =\mathbf {1}, \mathbf {y} = \mathbf {n} , a=-\gamma , b=\tau$ and $c=r$, and recalling from (3.4) that $r+\gamma =\mu _{{\tilde{D}}-2}\tau$, we have

3.14 $$\begin{aligned} \mathrm{e}^{ \varvec{{\varOmega }} x} \mathbf {1}=\mu _{{\tilde{D}}-2}^{-1} \left( \mathrm{e}^{rx} - \mathrm{e}^{-\gamma x} \right) \mathbf {n} + \mathrm{e}^{-\gamma x} \mathbf {1}\qquad \text{ and } \qquad \mathrm{e}^{ \varvec{{\varOmega }} x} \mathbf {n} = \mathrm{e}^{r x} \mathbf {n} . \end{aligned}$$

Thus,

3.15 $$\begin{aligned} \mathbf {m} (t)=\mu _{{\tilde{D}}-2}^{-1}\left( \mathrm{e}^{rt} - \mathrm{e}^{-\gamma t} \right) \mathbf {n} + \mathrm{e}^{-\gamma t}\mathbf {1}\end{aligned}$$

and

3.16 $$\begin{aligned} \lim _{t \rightarrow \infty }\mathrm{e}^{-r t} \mathbf {m} (t)=\mu _{{\tilde{D}}-2}^{-1} \mathbf {n} . \end{aligned}$$

While it is well known that asymptotically the mean prevalence grows exponentially with rate constant *r*, i.e. that $\mathrm {prevalence} \propto \mathrm{e}^{rt}$, these results allow us to see from (3.15) that the rate of convergence to this asymptotic behaviour is $r+\gamma$, and from (3.16) that the constant of proportionality is the degree of the initially infected individual divided by $\mu _{{\tilde{D}}-2}$.

We also consider the relationship between the equations above and the diverse ODE approaches to the mean behaviour of the full network epidemic model. Miller and Kiss (2014) consider several such approaches; their notation can be related to ours by defining

3.17 $$\begin{aligned} I(t) = \mathbf {m} ^{(k)}(t)^{\!\top } \mathbf {1} , \qquad \lambda (t) = \mathbf {m} ^{(k)}(t)^{\!\top } \mathbf {n} . \end{aligned}$$

Substituting (3.17) and (3.13) into (3.1) gives

3.18 $$\begin{aligned} \frac{\mathrm {d}I}{\mathrm {d}t} = \tau \lambda - \gamma I , \qquad \frac{\mathrm {d}\lambda }{\mathrm {d}t} = r \lambda . \end{aligned}$$

This pair of equations can be derived from various models considered by Miller and Kiss (2014, c.f. Sect. 3.4.1) assuming an initially small infectious population and negligible susceptible depletion. Therefore, our results suggest that the ODE approaches to mean behaviour do not require correction as the infectious population becomes extremely small, and the typical assumption that $1 \ll I(t=0) \ll N$ for the ODE system to hold may be too conservative, with $N \gg 1$ being all that is required.

## Variance

The variance in infectious prevalence during the exponentially growing phase of an epidemic was considered in Graham and House (2014), but using the diffusion limit and an argument about the neighborhood around an infective. A branching process limit lets us be more explicit. For $k\in \mathscr {K}$, let $\mathbf {u} _k$ denote the length-$(k_{\mathrm {max}}+1)$ column vector whose element corresponding to type *k* is 1 and all other elements are 0, so ${ \mathbf {u} _k}=(\delta _{i,k})$. For $t \ge 0$ and $k \in \mathscr {K}$, let $\sigma _{ij}^{(k)}(t)=\mathrm{cov}\left( Z^{(k)}_i(t),Z^{(k)}_j(t)\right)$ $(i,j \in \mathscr {K})$.

A matrix integrating factor argument gives

4.1 $$\begin{aligned} \varvec{V} ^{(k)}(t)=\left[ \sigma _{ij}^{(k)}(t)\right]&= \mathbb {E}\left[ \mathbf {Z} ^{(k)}(t) \mathbf {Z} ^{(k)}(t)^{\!\top }\right] -\mathbb {E}\left[ \mathbf {Z} ^{(k)}(t)\right] \mathbb {E}\left[ \mathbf {Z} ^{(k)}(t)^{\!\top }\right] \nonumber \\&= \int _{0}^{t} \mathrm{e}^{ \varvec{{\varOmega }} ^{\!\top }(t-u)} \varvec{B} _k(u) \mathrm{e}^{{ \varvec{{\varOmega }} }(t-u)} \mathrm {d}u , \end{aligned}$$

where

4.2 $$\begin{aligned} \varvec{B} _k(t)&= \sum _{l=0}^{k_{\mathrm {max}}} \left( \mathrm{e}^{ \varvec{{\varOmega }} t}\right) _{k,l} \varvec{C} _l ,\nonumber \\ \varvec{C} _k&= \omega _k \left( \varvec{\partial } ^2 P_{k}(\mathbf {1}) +\mathrm {diag}( \mathbf {f} _k) - \mathbf {u} _k \mathbf {f} _k^{\!\top }- \mathbf {f} _k \mathbf {u} _k^{\!\top }+ \mathbf {u} _k \mathbf {u} _k^{\!\top } \right) , \nonumber \\ \mathbf {f} _k&= \varvec{\partial } P_k(\mathbf {1}) . \end{aligned}$$

See Dorman et al. (2004, Sect. 9), and also Athreya and Ney (1972, p. 203), for details.^1^

For $t \ge 0$ and $k\in \mathscr {K}$, let $v^{(k)}(t)$ denote the variance of the total population size at time *t* given that the process starts with a single individual, whose type is *k*. Then $v^{(k)}(t)= \mathbf {1}^{\!\top } \varvec{V} ^{(k)}(t) \mathbf {1}$ and it follows using (4.1) that

4.3 $$\begin{aligned} v^{(k)}(t)&= \int _{0}^{t} (\mathrm{e}^{ \varvec{{\varOmega }} (t-u)} \mathbf {1})^{\!\top } \varvec{B} _k(u) (\mathrm{e}^{{ \varvec{{\varOmega }} }(t-u)} \mathbf {1}) \mathrm {d}u\nonumber \\&= \int _{0}^{t} \left( \mu _{{\tilde{D}}-2}^{-1} \left( \mathrm{e}^{r(t-u)} - \mathrm{e}^{-\gamma (t-u)} \right) \right) ^2 \mathbf {n} ^{\!\top } \varvec{B} _k(u) \mathbf {n} \; \mathrm {d}u \nonumber \\&\quad + 2 \int _{0}^{t} \mu _{{\tilde{D}}-2}^{-1} \left( \mathrm{e}^{r(t-u)} - \mathrm{e}^{-\gamma (t-u)} \right) \mathrm{e}^{-\gamma (t-u)} \mathbf {1} ^{\!\top } \varvec{B} _k(u) \mathbf {n} \; \mathrm {d}u\nonumber \\&\quad + \int _{0}^{t} \mathrm{e}^{-2 \gamma (t-u)} \mathbf {1} ^{\!\top } \varvec{B} _k(u) \mathbf {1} \; \mathrm {d}u , \end{aligned}$$

where we have used the first equation in (3.14) in deriving the last line. This quantity has an exact but rather complex closed-form solution, which we give below and derive in “Appendix 3”.

Let $\mathbf {n} _2=(0^2,1^2,\ldots ,{k_{\mathrm {max}}}^2)^{\!\top }$ and, for $t\ge 0$, let $\mathbf {v} (t)=(v^{(i)}(t))$. Then

4.4 $$\begin{aligned} \mathbf {v} (t)=\alpha _0(t) \mathbf {1}+ \alpha _1(t) \mathbf {n} + \alpha _2(t) \mathbf {n} _2, \end{aligned}$$

where

4.5 $$\begin{aligned} \alpha _0(t)&= \gamma I_2(t),\nonumber \\ \alpha _1(t)&= \gamma \mu _{{\tilde{D}}-2}^{-1}\left[ I_1(t)-I_2(t)+2I_3(t)+\mu _{{\tilde{D}}-2}^{-1}\mu _{{\tilde{D}}}^{-1}\mu _{({\tilde{D}}-1)^2+1}(I_4(t)-I_5(t))\right] \nonumber \\&\quad +\tau \left[ I_1(t)+2I_3(t)+\mu _{{\tilde{D}}-2}^{-2}\mu _{({\tilde{D}}-2)^2}I_4(t)\right] ,\nonumber \\ \alpha _2(t)&= \gamma \mu _{{\tilde{D}}-2}^{-2} I_5(t), \end{aligned}$$

with

4.6 $$\begin{aligned} I_1(t)&=\frac{\mathrm{e}^{rt}-\mathrm{e}^{-2\gamma t}}{r+2\gamma },\nonumber \\ I_2(t)&=\frac{\mathrm{e}^{-\gamma t}\left( 1-\mathrm{e}^{-\gamma t}\right) }{\gamma },\nonumber \\ I_3(t)&=\frac{\mathrm{e}^{rt}\left( 1-\mathrm{e}^{-\gamma t}\right) }{\gamma }-I_1(t),\nonumber \\ I_4(t)&=\frac{\mathrm{e}^{rt}\left( \mathrm{e}^{rt}-1\right) }{r}-2I_3(t)-I_1(t) ,\nonumber \\ I_5(t)&=\frac{\mathrm{e}^{2rt}-\mathrm{e}^{-(\gamma +2\tau )t}}{2r+2\tau +\gamma } -2\frac{\mathrm{e}^{-\gamma t}\left( \mathrm{e}^{rt}-\mathrm{e}^{-2\tau t}\right) }{r+2\tau }+\frac{\mathrm{e}^{-\gamma t}\left( \mathrm{e}^{-2\tau t}-\mathrm{e}^{-\gamma t}\right) }{\gamma -2\tau }. \end{aligned}$$

In (4.6), if a denominator is zero then the expression is given by the limit as that denominator tends to zero. For example, if $\gamma =0$ then $I_2(t)=t\mathrm{e}^{-\gamma t}$, and if $\gamma =2\tau$, the final term in $I_5(t)$ is replaced by $t\mathrm{e}^{-2 \gamma t}$.

Equation (4.4) leads to a rather complex expression for $\mathbf {v} (t)$ in terms of elementary functions. However, its asymptotic form as $t \rightarrow \infty$ is much simpler. Note that $\lim _{t \rightarrow \infty } \mathrm{e}^{-2rt} I_k(t)=0$, for $k=1,2,3$, $\lim _{t \rightarrow \infty } \mathrm{e}^{-2rt} I_4(t)=\frac{1}{r}$ and $\lim _{t \rightarrow \infty } \mathrm{e}^{-2rt} I_5(t)=\frac{1}{2r+2\tau +\gamma }=\frac{1}{2\mu _{{\tilde{D}}-1}\tau -\gamma }$. Substituting these limits into (4.4) and (4.5) yields

4.7 $$\begin{aligned} \lim _{t \rightarrow \infty } \mathrm{e}^{-2rt} \mathbf {v} (t)= \frac{1}{\mu _{{\tilde{D}}-2}^2\left( 2\mu _{{\tilde{D}}-1}\tau -\gamma \right) } \left[ \frac{2\tau \mu _{{\tilde{D}}-1}\left( \mu _{({\tilde{D}}-2)^2} \tau +\gamma \right) }{r} \mathbf {n} +\gamma \mathbf {n} _2\right] .\nonumber \\ \end{aligned}$$

Note that both the asymptotic and exact expressions for $\mathbf {v} (t)$ depend on the degree distribution *D* only through its first three moments.

It follows from (3.16) and (4.7) that, for $k\in \mathscr {K}$,

4.8 $$\begin{aligned} \lim _{t \rightarrow \infty }\frac{\mathrm{var}\left( Z^{(k)}(t)\right) }{\mathbb {E}\left[ Z^{(k)}(t)\right] ^2}= \frac{1}{2\mu _{{\tilde{D}}-1}\tau -\gamma } \left[ \gamma +\frac{2\tau \mu _{{\tilde{D}}-1}\left( \mu _{({\tilde{D}}-2)^2} \tau +\gamma \right) }{kr}\right] . \end{aligned}$$

We note two features of these results. First, the Eqs. (4.4) and (4.5) involve many rates that are linear combinations of *r*, $\tau$ and $\gamma$, with the dominant being 2*r* and the subdominant being *r*. This leads to complex real-time behaviour as the system approaches its asymptotic limit. In the diffusion limit, only the dominant and subdominant rates are present, leading to the same overall rate of convergence *r*, but other rates are not present (Graham and House 2014). Secondly, the dependence of the variance on initial conditions is not simple proportionality, meaning that (4.7) contains terms proportional to both $\mathbf {n}$ and $\mathbf {n} _2$ (unless $\gamma =0$) and the right-hand side of (4.8) depends on *k*.

## Covariance function

For $t,s \ge 0$ and $k\in \mathscr {K}$, let $\sigma ^{(k)}(t,s)=\mathrm{cov}\left( Z^{(k)}(t), Z^{(k)}(s)\right)$ denote the covariance of the total population sizes at times *t* and *s* in the branching process which approximates the early phase of an epidemic, given that the process starts with a single individual, whose type is *k*. We assume without loss of generality that $t \le s$; although this choice does not respect alphabetical order, the majority of results that follow take *t* as an argument rather than *s*, and are therefore more easily read as functions of time. Then

5.1 $$\begin{aligned} \sigma ^{(k)}(t,s)&=\mathbb {E}\left[ \mathrm{cov}\left( Z^{(k)}(t), Z^{(k)}(s)|\mathbf {Z}^{(k)}(t)\right) \right] \nonumber \\&\quad +\mathrm{cov}\left( \mathbb {E}\left[ Z^{(k)}(t)|\mathbf {Z}^{(k)}(t)\right] ,\mathbb {E}\left[ Z^{(k)}(s)|\mathbf {Z}^{(k)}(t)\right] \right) . \end{aligned}$$

The first term on the right hand side of (5.1) is zero, since $Z^{(k)}(t)$ is non-random given $\mathbf {Z}^{(k)}(t)$. Now, $\mathbb {E}\left[ Z^{(k)}(t)|\mathbf {Z}^{(k)}(t)\right] = \mathbf {1} ^{\!\top }\mathbf {Z}^{(k)}(t)$ and $\mathbb {E}\left[ Z^{(k)}(s)|\mathbf {Z}^{(k)}(t)\right] =\mathbf {Z}^{(k)}(t)^{\!\top } \varvec{M} (s-t)\mathbf {1}$, so

5.2 $$\begin{aligned} \sigma ^{(k)}(t,s)&=\mathrm{cov}\left( \mathbf {1} ^{\!\top }\mathbf {Z}^{(k)}(t),\mathbf {Z}^{(k)}(t)^{\!\top } \varvec{M} (s-t)\mathbf {1}\right) \nonumber \\&= \mathbf {1} ^{\!\top } \varvec{V} ^{(k)}(t) \varvec{M} (s-t)\mathbf {1}\nonumber \\&=\mu _{{\tilde{D}}-2}^{-1}\left( \mathrm{e}^{r(s-t)}-\mathrm{e}^{-\gamma (s-t)}\right) \mathbf {1} ^{\!\top } \varvec{V} ^{(k)}(t) \mathbf {n} +\mathrm{e}^{-\gamma (s-t)}v^{(k)}(t), \end{aligned}$$

using (3.2), the first equation in (3.14) and noting that $\mathbf {1} ^{\!\top } \varvec{V} ^{(k)}(t) \mathbf {1} =v^{(k)}(t)$. This leads to an exact, closed-form expression for the covariance function in terms of elementary functions, which we state below and derive in “Appendix 4”.

For $t,s\ge 0$, let $\mathbf {\sigma } (t,s)=\left( \sigma ^{(0)}(t,s),\sigma ^{(1)}(t,s),\ldots ,\sigma ^{(k_{\mathrm {max}})}(t,s)\right) ^{\!\top }$. Then

5.3 $$\begin{aligned} \mathbf {\sigma } (t,s)=\mu _{{\tilde{D}}-2}^{-1}\left( \mathrm{e}^{r(s-t)}-\mathrm{e}^{-\gamma (s-t)}\right) \left( \beta _1(t) \mathbf {n} + \beta _2(t) \mathbf {n} _2\right) +\mathrm{e}^{-\gamma (s-t)} \mathbf {v} (t), \end{aligned}$$

where

5.4 $$\begin{aligned} \beta _1(t)&= \gamma \mu _{{\tilde{D}}-2}^{-1}\left[ \mu _{{\tilde{D}}}^{-1}\mu _{({\tilde{D}}-1)^2+1}(I_7(t)-I_8(t))+\mu _{{\tilde{D}}-2}I_6(t)\right] \nonumber \\&\quad +\tau \mu _{{\tilde{D}}-2}^{-1}\left[ \mu _{({\tilde{D}}-2)^2}I_7(t)+ \mu _{{\tilde{D}}-2}^2 I_6(t)\right] ,\nonumber \\ \beta _2(t)&= \gamma \mu _{{\tilde{D}}-2}^{-1} I_8(t), \end{aligned}$$

with

5.5 $$\begin{aligned} I_6(t)&=\frac{\mathrm{e}^{rt}\left( 1-\mathrm{e}^{-\gamma t}\right) }{\gamma } ,\nonumber \\ I_7(t)&=\frac{\mathrm{e}^{rt}\left( \mathrm{e}^{rt}-1\right) }{r}-I_6(t) ,\nonumber \\ I_8(t)&=\frac{\mathrm{e}^{2rt}-\mathrm{e}^{-(\gamma +2\tau )t}}{2r+2\tau +\gamma }-\frac{\mathrm{e}^{-\gamma t}\left( \mathrm{e}^{rt}-\mathrm{e}^{-2\tau t}\right) }{r+2\tau }. \end{aligned}$$

As at (4.6), an appropriate limit is taken if a denominator in (5.5) is zero.

The covariance function takes a simple form in the limit as *t* and $s \rightarrow \infty$. Note that $\lim _{t \rightarrow \infty } \mathrm{e}^{-2rt} I_6(t)=0$, $\lim _{t \rightarrow \infty } \mathrm{e}^{-2rt} I_7(t)=\frac{1}{r}$ and $\lim _{t \rightarrow \infty } \mathrm{e}^{-2rt} I_8(t)=\frac{1}{2\mu _{{\tilde{D}}-1}\tau -\gamma }$. Substituting these limits into (5.3) yields that, for any $s\ge 0$,

5.6 $$\begin{aligned} \lim _{t \rightarrow \infty } \mathrm{e}^{-2rt} \mathbf {\sigma } (t,t+s)=\mathrm{e}^{rs} \lim _{t \rightarrow \infty } \mathrm{e}^{-2rt} \mathbf {v} (t). \end{aligned}$$

It follows that, for $k\in \mathscr {K}$ and $s>0$,

$$\begin{aligned} \lim _{t \rightarrow \infty } \mathrm{corr}\left( Z^{(k)}(t),Z^{(k)}(t+s)\right) =1, \end{aligned}$$

where $\mathrm{corr}$ denotes correlation. This is not surprising since it is well known that

$$\begin{aligned} \mathrm{e}^{-rt} Z^{(k)}(t) \xrightarrow {\text {a.s.}} W^{(k)} \quad \text {as } t \rightarrow \infty , \end{aligned}$$

where $\xrightarrow {\text {a.s.}}$ denotes almost sure convergence (i.e. convergence with probability 1) and $W^{(k)}$ is a non-negative random which satsifies $W^{(k)}=0$ if and only if the branching process goes extinct; see e.g.  Athreya and Ney (1972, Theorem V.7.2).

## Unbounded degree distributions

The above results have all assumed that there is a maximum degree $k_{\mathrm {max}}$. Suppose that is not the case, so the branching process has countably many types. For $t\ge 0$, let $\mathbf {Z}(t)=(Z_0(t), Z_1(t),\ldots )^{\!\top }$, where $Z_i(t)$ denotes the number of individuals of type *i* alive at time *t*, and let $Z(t)=\sum _{i=0}^\infty Z_i(t)$ denote the total number individuals alive at time *t*. (For ease of notation we drop explict reference to the type of the initial individual.) For $k_{\mathrm {max}}=1,2,\ldots$, let $\{\mathbf {Z}(t,k_{\mathrm {max}}):t \ge 0\}$ denote the branching process derived from $\{\mathbf {Z}(t):t \ge 0\}$ by ignoring all individuals having type strictly greater than $k_{\mathrm {max}}$ and any offspring of such individuals. For $t \ge 0$, let $Z(t, k_{\mathrm {max}})=\mathbf {1}^{\!\top }\mathbf {Z}(t,k_{\mathrm {max}})$ be the total number of individuals alive in $\{\mathbf {Z}(t,k_{\mathrm {max}}):t \ge 0\}$ at time *t*. Now, for any $t \ge 0$, $Z(t, k_{\mathrm {max}})$ is monotonically increasing in $k_{\mathrm {max}}$ and converges almost surely to *Z*(*t*) as $k_{\mathrm {max}}\rightarrow \infty$. Thus, by the monotone convergence theorem, $\mathbb {E}\left[ Z(t)\right] =\lim _{k_{\mathrm {max}}\rightarrow \infty }\mathbb {E}\left[ Z(t,k_{\mathrm {max}})\right]$.

The process $\{\mathbf {Z}(t,k_{\mathrm {max}}):t \ge 0\}$ behaves like the branching process described in Sect. 2.3 but with infection rate $\tau$ replaced by $\tau (k_{\mathrm {max}})=\tau \mathbb {P}\left( {\tilde{D}} \le k_{\mathrm {max}}+1\right)$, and size-biased degree distribution ${\tilde{D}}$ replaced by ${\tilde{D}}(k_{\mathrm {max}})$, where

$$\begin{aligned} \mathbb {P}\left( {\tilde{D}}(k_{\mathrm {max}})=k\right) = {\left\{ \begin{array}{ll} \frac{{\tilde{p}}_k}{\mathbb {P}\left( {\tilde{D}} \le k_{\mathrm {max}}+1\right) } &{} \text { if } k=1,2,\ldots ,k_{\mathrm {max}}+1 ,\\ 0 &{} \text { if } k= k_{\mathrm {max}}+2,k_{\mathrm {max}}+3,\ldots . \end{array}\right. } \end{aligned}$$

The presence of $k_{\mathrm {max}}+1$ rather than $k_{\mathrm {max}}$ is because contacts with individuals having degree strictly greater than $k_{\mathrm {max}}\!+\!1$ are ignored, as they yield individuals with effective degree (and hence type) strictly greater than $k_{\mathrm {max}}$. Now $\tau (k_{\mathrm {max}}) \!\rightarrow \! \tau$ and $\mathbb {E}\left[ {\tilde{D}}(k_{\mathrm {max}})\right] \!\!\rightarrow \!\! \mathbb {E}\left[ {\tilde{D}}\right]$ as $k_{\mathrm {max}}\!\!\rightarrow \!\! \infty$, so the expression (3.15) for the mean total population size at time *t* continues to hold in the unbouded degree case, provided that $\mathbb {E}\left[ {\tilde{D}}\right] \!<\!\infty$, or equivalently that $\mathbb {E}\left[ D^2\right] \!<\!\infty$. A similar argument shows that the expressions for the variance of *Z*(*t*) and the covariance of *Z*(*t*) and *Z*(*s*), derived in Sects. 4 and 5, respectively, continue to hold provided $\mathbb {E}\left[ {\tilde{D}}^2\right] \!<\!\infty$, or equivalently $\mathbb {E}\left[ D^3\right] \!<\!\infty$.

## Probability of extinction

For $t \ge 0$ and $k\in \mathscr {K}$, let $\pi _k(t)\!=\!\mathbb {P}\left( Z^{(k)}(t)\!=\!0\right)$ be the probability that the branching process is extinct at time *t* given that it starts with one individual of type *k*. Then in general

7.1 $$\begin{aligned} \frac{\mathrm {d}}{\mathrm {d}t}\pi _k(t) = -\omega _k \pi _k(t) + \omega _k P_k( \varvec{\pi } (t)) , \end{aligned}$$

where $\varvec{\pi } (t)=(\pi _i(t))$. For our specific model, using (2.1) and (2.3), we have

7.2 $$\begin{aligned} \frac{\mathrm {d}}{\mathrm {d}t}\pi _k(t) = -(\gamma + \tau k) \pi _k(t) + \gamma + \tau k \pi _{k-1}(t) \sum _{l=0}^{k_{\mathrm {max}}-1} {\tilde{p}}_{l+1} \pi _l(t) . \end{aligned}$$

These equations are not amenable to closed-form solution. Note, however, that studies of time to extinction for network epidemics—e.g. Holme (2013)—have tended to be based on Monte Carlo methods, but (7.2) could provide a complementary approach that is numerically cheaper and more analytically tractable.

We will now consider three regimes in which asymptotic methods can be used to bound the real-time behaviour of the probabilities of extinction. In particular, we will see that early real-time behaviour is bounded by the death rates $\omega _k$, while late-time behaviour is bounded by the asymptotic real-time growth rate *r* provided $r>-\gamma$.

### Late behaviour of the subcritical case

Suppose that $r<0$, so the branching process is subcritical. For $t \ge 0$ and $k\in \mathbb {N}_0=\{0,1,\ldots \}$, we will work with the probability of survival $q_k(t)=1-\pi _k(t)=\mathbb {P}\left( Z^{(k)}(t)>0\right)$. Now $\mathbb {P}\left( Z^{(k)}(t)>0\right) \le \mathbb {E}\left[ Z^{(k)}(t)\right]$, so using (3.15) a simple upper bound for $q_k(t)$, valid also in the unbounded degree setting using the results in Sect. 6, is

7.3 $$\begin{aligned} q_k(t) \le k\mu _{{\tilde{D}}-2}^{-1}\left( \mathrm{e}^{rt} - \mathrm{e}^{-\gamma t} \right) + \mathrm{e}^{-\gamma t} . \end{aligned}$$

Note that $\mu _{{\tilde{D}}} < \infty$ is a necessary condition for $r<0$. Under the stronger condition that $\mu _{{\tilde{D}}^2}< \infty$, Windridge (2015) gives an exponential approximation, for large *t*, to a quantity closely related to $q_k(t)$. He assumes that what we call type-0 individuals are dead. For $k=1,2,\ldots$, let ${\hat{q}}_k(t)=\mathbb {P}\left( \sum _{i=1}^\infty Z_i^{(k)}(t)>0\right)$. Then, Windridge shows that there exists a constant ${\hat{c}} \in (0,1]$ such that, for any $a < \min \{\tau , -r\}$,

7.4 $$\begin{aligned} {\hat{q}}_k(t) ={\hat{c}} k \mathrm{e}^{rt}\left( 1+O(k\mathrm{e}^{-at})\right) \quad \text{ as } t \rightarrow \infty , \end{aligned}$$

for any $k \ge 1$. The constant ${\hat{c}}=\lim _{t\rightarrow \infty }\mathrm{e}^{-r t} {\hat{q}}_1(t)$. Note that for some practical purposes, ${\hat{q}}_k(t)$ may be of more interest than $q_k(t)$, since type-0 individuals are unable to transmit infection. In particular, in “Appendix 5” we sketch the argument that for the case where $r>-\gamma$ an analogous result to (7.4) holds, i.e. , for $k \ge 1$,

7.5 $$\begin{aligned} {q}_k(t)\sim c k \mathrm{e}^{rt} \quad \text {as}\, t \rightarrow \infty ,\quad \text {where} \, {c}=\lim _{t\rightarrow \infty }\mathrm{e}^{-r t} q_1(t)>0 . \end{aligned}$$

(For real-valued functions, *f* and *g* say, $f(t) \sim g(t)$ as $t \rightarrow \infty$ if $\lim _{t \rightarrow \infty } f(t)/g(t)=1$.)

For the case where $r<-\gamma$ (so, from (3.4), $\mu _{{\tilde{D}}}<2$) we show in “Appendix 5” that if $\mu _{{\tilde{D}}^2}< \infty$ then, for $k \ge 0$,

7.6 $$\begin{aligned} {q}_k(t)\sim (1-k\mu _{{\tilde{D}}-2}^{-1})\mathrm{e}^{- \gamma t} \quad \text {as}\, t \rightarrow \infty . \end{aligned}$$

Note that in this case the asymptotic behaviour of the survival probability ${q}_k(t)$ is independent of the infection rate $\tau$. The case $r<-\gamma$ could occur, for example, at the end of an epidemic where $\tau \gg \gamma$. Such an epidemic would consist primarily of transmission events at early times, with the late behaviour dominated by recovery events.

### Late behaviour of the supercritical case

An approximation to $q_k(t)$ in the supercritical case ($r>0$) can be obtained by exploiting the fact that a supercritical branching process conditioned on extinction is probabilistically equivalent to a subcritical branching process. For $k\in \mathbb {N}_0$, let $T^{(k)}=\inf \{t \ge 0:Z^{(k)}(t)=0\}$ denote the extinction time of the branching process given that it starts with one individual of type *k*, where $T^{(k)}=\infty$ if the branching process survives forever, and let $\pi _k=\mathbb {P}\left( T^{(k)}<\infty \right) =\pi _k(\infty )$ be the probability that the branching process ultimately goes extinct. Then,

7.7 $$\begin{aligned} q_k(t)=1-\pi _k+\pi _k \mathbb {P}\left( T^{(k)}>t|T^{(k)}<\infty \right) . \end{aligned}$$

Let $\{\tilde{ \mathbf {Z} }^{(k)}(t):t \ge 0 \}$ be distributed as $\{ \mathbf {Z} ^{(k)}(t):t \ge 0 |T^{(k)}<\infty \}$. Then it follows from  Waugh (1958, Sect. 5), that $\{\tilde{ \mathbf {Z} }^{(k)}(t):t \ge 0 \}$ is also a continuous-time multitype Markov branching process, in which the lifetime of a typical type-*k* individual has an exponential distribution with rate $\gamma +\tau k$, as at (2.1), but when it dies its offspring is now distributed as follows:

7.8 $$\begin{aligned} \mathbb {P}\left( \mathrm {Offspring} = \varnothing | \mathrm {Parent\ type} = k \right)&= \frac{1}{\pi _k}\frac{\gamma }{\gamma + \tau k} , \nonumber \\ \mathbb {P}\left( \mathrm {Offspring} = \{k-1,l\} | \mathrm {Parent\ type} = k \right)&= \frac{\pi _{k-1} \pi _l}{\pi _k}\frac{\tau k {\tilde{p}}_{l+1}}{\gamma + \tau k} \quad (l\in \mathbb {N}_0) . \end{aligned}$$

Suppose now that there is a maximum degree size $k_{\mathrm {max}}$. Let $\tilde{ \varvec{{\varOmega }} }=[\tilde{{\varOmega }}_{l,k}]$ be the $(k_{\mathrm {max}}+1) \times (k_{\mathrm {max}}+1)$ matrix with elements given by

$$\begin{aligned} \tilde{{\varOmega }}_{l,k} = \frac{\pi _{l-1}\pi _k}{\pi _l}\tau l \left( {\tilde{p}}_{k+1} + \delta _{l,k+1}\right) -(\gamma + \tau l) \delta _{l,k} \qquad (l,k\in \mathscr {K}). \end{aligned}$$

Then, recalling (3.2),

7.9 $$\begin{aligned} \mathbb {E}\left[ {\tilde{Z}}^{(k)}(t)\right] = \mathbf {u} _k^{\!\top }\mathrm{e}^{\tilde{ \varvec{{\varOmega }} }t} \mathbf {1}, \end{aligned}$$

where $${\tilde{Z}}^{(k)}(t)={\tilde{Z}}^{(k)}_0(t)+{\tilde{Z}}^{(k)}_1(t)+\ldots +{\tilde{Z}}^{(k)}_{k_{\mathrm {max}}}(t)$$.

Let $\tilde{r}$ denote the dominant eigenvalue of $\tilde{ \varvec{{\varOmega }} }$ and note that $\tilde{r}<0$. For $t \ge 0$ and $k=0,1,\ldots , k_{\mathrm {max}}$, let ${\tilde{q}}_k(t)=\mathbb {P}\left( {\tilde{Z}}^{(k)}(t)>0\right)$ be the probability that the branching process $\{\tilde{ \mathbf {Z} }^{(k)}(t):t \ge 0 \}$ is not extinct at time *t* given that it starts with one individual of type *k*. Then we expect that arguments similar to those used in the proof of Heinzmann (2009, Theorem 3.1), will show that there exists constants $\tilde{c}_1, \tilde{c}_2,\ldots ,\tilde{c}_{k_{\mathrm {max}}}$, satisfying $0<\tilde{c}_k<\infty$, such for $k=1,2,\ldots ,k_{\mathrm {max}}$,

7.10 $$\begin{aligned} {\tilde{q}}_k(t) =\tilde{c}_k \mathrm{e}^{\tilde{r}t}\left( 1+o(\mathrm{e}^{-\tilde{\gamma }t})\right) \quad \text{ as } t \rightarrow \infty , \end{aligned}$$

for any $\tilde{\gamma }>0$. It then follows using (7.7) that

7.11 $$\begin{aligned} q_k(t)=1-\pi _k+\pi _k \tilde{c}_k \mathrm{e}^{\tilde{r}t}\left( 1+o(\mathrm{e}^{-\tilde{\gamma }t})\right) \quad \text{ as } t \rightarrow \infty . \end{aligned}$$

Heinzmann (2009, Theorem 3.1), cannot be applied directly as it assumes that the matrix $\tilde{ \varvec{{\varOmega }} }$ is irreducible. We do not consider it here but we expect that Heinzmann’s proof can be extended to our situation. If we assume that type-0 individuals are dead and only consider initial individuals of types $1,2,\ldots ,k_{\mathrm {max}}-1$ (recall that only initial infectives can have type $k_{\mathrm {max}}$) then $\tilde{ \varvec{{\varOmega }} }$ becomes a $(k_{\mathrm {max}}-1) \times (k_{\mathrm {max}}-1)$ irreducible matrix. Heinzmann (2009, Theorem 3.1), then yields (7.10); note that now $\pi _k$ is replaced by ${\bar{\pi }}_k=1-\lim _{t \rightarrow \infty } {\bar{q}}_k(t)$ $(k=1,2,k_{\mathrm {max}}-1)$ in the definition of $\tilde{ \varvec{{\varOmega }} }$ and ${\tilde{q}}_k(t)={\tilde{q}}_k(t)=\mathbb {P}\left( \sum _{i=1}^{k_{\mathrm {max}}-1}{\tilde{Z}}^{(k)}_i(t)>0\right)$. The approximation (7.11) then holds with $q_k(t)$ and $\pi _k$ replaced by ${\bar{q}}_k(t)$ and ${\bar{\pi }}_k$, repsectively. Moreover, if we then let $\tilde{ \mathbf {f} }_1^{\!\top }$ and $\tilde{ \mathbf {b} }_1$ be left and right eigenvectors of $\tilde{ \varvec{{\varOmega }} }$ corresponding to the eigenvalue $\tilde{r}$, satisfying $\tilde{ \mathbf {f} }_1^{\!\top }\tilde{ \mathbf {b} }_1=1$, then $\tilde{c}_k=\left( \mathbf {u} _k^{\!\top }\tilde{ \mathbf {b} }_1\right) h^*$, where $h^*=\lim _{t \rightarrow \infty } \mathrm{e}^{-\tilde{r}t}\tilde{ \mathbf {f} }_1^{\!\top }\tilde{ \mathbf {q} }(t)$ and $\tilde{ \mathbf {q} }(t)=\left( {\tilde{q}}_i(t),{\tilde{q}}_2(t), \ldots , {\tilde{q}}_{k_{\mathrm {max}}-1}(t)\right) ^{\!\top }$. Unfortunately, unlike with $\varvec{{\varOmega }}$, there do not appear to be closed-form expressions for $\tilde{r}$ and its associated eigenvectors.

### Early behaviour and matched asymptotics

Matched asymptotics is a standard technique in mathematical biology for writing down approximations to non-linear models that match known asymptotic behaviour (Murray 2002, 2003). While numerical solution of the ODEs (7.2) is efficient (as we have noted above) we now obtain a crude approximation to the full system that takes a closed form in terms of elementary functions.

First note that for $k\in \mathscr {K}$, $\pi _k(0) = 0$ and $\pi _k(t)$ is monotonically increasing with *t*. If we neglect the quadratic terms in $\pi$ in (7.2) then, since these are only positive and increasing over time, we get a lower bound for the extinction probabilities:

7.12 $$\begin{aligned} \pi _k(t) \ge \pi _k^{(0)}(t)= \frac{\gamma }{\omega _k} \left( 1 - \mathrm{e}^{-\omega _k t} \right) . \end{aligned}$$

Note that in standard matched asymptotics, we would identify a small parameter from a ratio of rate constants as the basis for a systematic approximation scheme (Murray 1984); an alternative would be to approximate systematically by, for example, letting $\pi _k^{(1)} = \pi _k - \pi _k^{(0)}$, substituting into (7.2) and neglecting quadratic terms to give a linear set of equations for the next order of approximation. Here we consider only the lowest order approximation, and hence define an ‘internal’ solution for the survival probability as:

7.13 $$\begin{aligned} q_k^{(I)}(t) = 1-\pi _k^{(0)}(t) =\frac{1}{\omega _k} \left( \tau k + \gamma \mathrm{e}^{-\omega _k t} \right) . \end{aligned}$$

Next, supposing we are in the subcritical case so that our result (7.5) holds. We will call this the ‘external’ solution

7.14 $$\begin{aligned} q_k^{(E)}(t) = ck\mathrm{e}^{r t}. \end{aligned}$$

To fix the constant *c*, we match the late behaviour of the internal solution with the early behaviour of the external solution:

7.15 $$\begin{aligned} \overline{q} = \lim _{t\rightarrow \infty } q_k^{(I)}(t) = \lim _{t\rightarrow 0} q_k^{(E)}(t) \quad \Rightarrow \quad q_k^{(E)}(t) = \frac{\tau k}{\omega _k} \mathrm{e}^{r t} . \end{aligned}$$

Finally, the matched asymptotic solution is

7.16 $$\begin{aligned} q_k^{(A)}(t) = q_k^{(I)}(t) + q_k^{(E)}(t) - \overline{q} = \frac{1}{\omega _k} \left( \tau k \mathrm{e}^{r t} + \gamma \mathrm{e}^{-\omega _k t} \right) . \end{aligned}$$

**Fig. 1:**
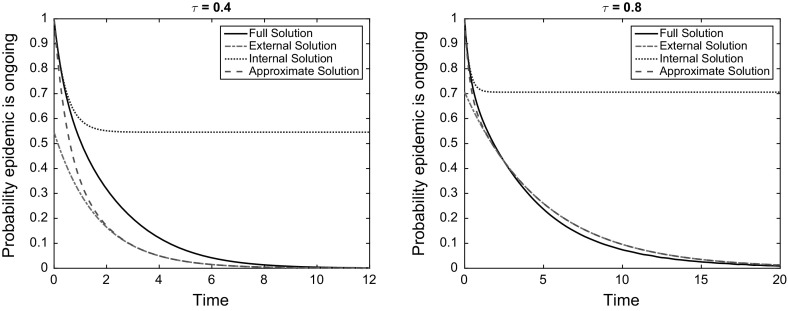
Extinction probability for a subcritical epidemic compared to approximations. Results are for a 3-regular graph with $\gamma = 1$ and values of $\tau$ indicated in the figure titles. The internal and external solutions each fail severely at certain points, but the approximate solution crudely captures the overall behaviour (colour figure online)

We compared this approximation as well as the internal and external solutions to the exact solution $q_k(t)$, with results shown in Fig. 1. As advertised, this is a relatively crude approximation, but is expressed in terms of elementary functions and satisfies known asymptotic limits.

## Fluctuations in the emerging phase of a major outbreak

We now consider the early behaviour of supercritical epidemics that take off (i.e. do not go extinct early on but ultimately end owing to long-term depletion of susceptibles). The early stages of such an epidemic are approximated by the branching process defined in Sect. 2.3 but conditioned on non-extinction. It is straightforward to adapt the results on means and variances in Sects. 3 and 4 to condition on $Z^{(k)}(t)>0$. Elementary calculation shows that, for $t \ge 0$ and $k\in \mathbb {N}_0$,

$$\begin{aligned} \mathbb {E}\left[ Z^{(k)}(t)\Big |Z^{(k)}(t)>0\right]&=\frac{\mathbb {E}\left[ Z^{(k)}(t)\right] }{q_k(t)},\\ \mathrm{var}\left( Z^{(k)}(t)\Big |Z^{(k)}(t)>0\right)&=\frac{\mathrm{var}\left( Z^{(k)}(t)\right) }{q_k(t)}-\pi _k(t)\left( \frac{\mathbb {E}\left[ Z^{(k)}(t)\right] }{q_k(t)}\right) ^2. \end{aligned}$$

Expressions for $\mathbb {E}\left[ Z^{(k)}(t)\Big |Z^{(k)}(t)>0\right]$ and $\mathrm{var}\left( Z^{(k)}(t)\Big |Z^{(k)}(t)>0\right)$ then follow using (3.15) and (4.4), respectively, though there is no closed-form formula for $q_k(t)$ or $\pi _k(t)$. Note that, assuming $r>0$ so $\pi _k<1$,

$$\begin{aligned} \lim _{t \rightarrow \infty }\frac{\mathrm{var}\left( Z^{(k)}(t)\Big |Z^{(k)}(t)>0\right) }{\mathbb {E}\left[ Z^{(k)}(t)\Big |Z^{(k)}(t)>0\right] ^2} =\left( 1-\pi _k\right) \lim _{t \rightarrow \infty }\frac{\mathrm{var}\left( Z^{(k)}(t)\right) }{\mathbb {E}\left[ Z^{(k)}(t)\right] ^2}-\pi _k , \end{aligned}$$

which depends on the degree *k* of the initial infective.

The diffusion approximation studied in Graham and House (2014) corresponds to the case where the number of infectives at time $t=0$ is large. Return to the case where there is a maximum degree $k_{\mathrm {max}}$ and suppose that the branching process does not go extinct. Then it follows from Athreya and Ney (1972, Theorem V.7.2), that, for any $k\in \mathscr {K}$,

$$\begin{aligned} \frac{ \mathbf {Z} ^{(k)}(t)}{Z^{(k)}(t)} \xrightarrow {\text {a.s.}} \mathbf {w} _1 \quad \text {as } t \rightarrow \infty , \end{aligned}$$

where $\mathbf {w} _1$, given by (3.10), is a left eigenvector of $\varvec{{\varOmega }}$ corresponding to the dominant eigenvalue $\lambda _1=r$. Thus if an epidemic takes off and is still in its exponentiallly growing phase then the relative frequencies of the different types of infectives will be close to $\mathbf {w} _1$. Hence, we now assume that the initial number of individuals in the branching process $Z(0)=K$, where *K* is large, and that $Z_i(0) \approx w_{1,i} K$ for $i \in \mathscr {K}$. Label the initial individuals $1,2,\ldots ,K$. Then, for $t \ge 0$, the total population size is $Z(t)={\hat{Z}}_1(t)+ {\hat{Z}}_2(t)+\ldots +{\hat{Z}}_K(t)$, where ${\hat{Z}}_i(t)$ denotes the total number of descentants of the initial individual *i* that are alive at time *t*, including *i* itself if it is still alive. Thus, $\mathbb {E}\left[ Z(t)\right] =\sum _{i=1}^K \mathbb {E}\left[ {\hat{Z}}_i(t)\right]$, for all $t \ge 0$, and, since the processes $\{{\hat{Z}}_i(t):t \ge 0\}$ $(i=1,2,\ldots ,K)$ are mutually independent, $\mathrm{var}\left( Z(t)\right) =\sum _{i=1}^K \mathrm{var}\left( {\hat{Z}}_i(t)\right)$, for all $t \ge 0$, and $\mathrm{cov}\left( Z(t), Z(s)\right) = \sum _{i=1}^K \mathrm{cov}\left( {\hat{Z}}_i(t), {\hat{Z}}_i(s)\right)$, for all $t,s \ge 0$.

Note that (3.9) implies that

$$\begin{aligned} \mathbf {w} _1^{\!\top } \mathbf {n} =\sum _{i=0}^{k_{\mathrm {max}}} i w_{1,i}=\mu _{{\tilde{D}}-2} \qquad \text{ and }\qquad \mathbf {w} _1^{\!\top } \mathbf {n} _2= \sum _{i=0}^{k_{\mathrm {max}}} i^2 w_{1,i}=\frac{\mu _{{\tilde{D}}-2} \mu _{({\tilde{D}}-1)^2+1}}{\mu _{{\tilde{D}}}} . \end{aligned}$$

Assuming that the above approximation is exact, then, for $t \ge 0$, it follows from (3.15) that

8.1 $$\begin{aligned} \mathbb {E}\left[ Z(t)\right] =K \mathbf {w} _1^{\!\top } \mathbf {m} (t)=K \mathrm{e}^{rt} \end{aligned}$$

and, after a little algebra, it follows from (4.4) that

8.2 $$\begin{aligned} \mathrm{var}\left( Z(t)\right)&= K \mathbf {w} _1^{\!\top } \mathbf {v} (t)\nonumber \\&=K\left( \gamma \left[ I_9(t)+\mu _{{\tilde{D}}}^{-1}\mu _{{\tilde{D}}-2}^{-1}\left( \sigma _{{\tilde{D}}}^2+2\right) I_4(t)\right] \right. \nonumber \\&\quad \left. + \tau \mu _{{\tilde{D}}-2}^{-1}\left[ \mu _{{\tilde{D}}-2}^2 I_9(t)+\sigma _{{\tilde{D}}}^2 I_4(t)\right] \right) , \end{aligned}$$

where $$\sigma _{{\tilde{D}}}^2=\mathrm{var}({\tilde{D}})$$ and

$$\begin{aligned} I_9(t)=\frac{\mathrm{e}^{rt}\left( \mathrm{e}^{rt}-1\right) }{r}. \end{aligned}$$

Comparison of (8.1) and (8.2) with the diffusion-based result of Graham and House (2014) in the limit of large *t* gives agreement when $\gamma = 0$ (i.e. for the SI model) but not for $\gamma >0$. We believe that this is due to the fact that the diffusion model was only four dimensional, so a heuristic argument (given in Sect. 3.3 of Graham and House 2014, which gave results that were in good agreement with simulation) about the neighbourhood of an infective node had to be made, in contrast to the approach here that deals with each effective degree explicitly and so has $k_{\mathrm {max}}+1$ dimensions. The argument about the neighbourhood around an infective tries to account for correlations caused by variability in recovery times, and so if $\gamma \rightarrow 0$ then these correlations do not exist.

Recent work by Constable and McKane (2014) considered the reduction of high-dimensional stochastic models to low-dimensional diffusions and this approach was shown to be asymptotically exact for some systems in the small-noise limit by Parsons and Rogers (2015). It is an open question whether the argument in Sect. 3.3 of Graham and House (2014) could be justified rigorously by a similar argument, however we note that a branching-process approach makes fewer assumptions than a low-dimensional diffusion limit and so will be more generally applicable.

Considering further results that can be obtained, it follows from (5.3) and a little algebra that, for $0 \le t \le s$,

8.3 $$\begin{aligned} \mathrm{cov}\left( Z(t), Z(s)\right)&=\mathrm{e}^{-\gamma (s-t)} \mathrm{var}\left( Z(t)\right) +K\mu _{{\tilde{D}}-2}^{-1}\left( \mathrm{e}^{r(s-t)}-\mathrm{e}^{-\gamma (s-t)}\right) \nonumber \\&\quad \times \left\{ \gamma \mu _{{\tilde{D}}}^{-1}\left[ \mu _{({\tilde{D}}-1)^2+1}I_9(t)- \left( \sigma _{{\tilde{D}}}^2+2\right) I_6(t)\right] \right. \nonumber \\&\quad +\left. \tau \left[ \mu _{{\tilde{D}}-2}^2 I_9(t)-\sigma _{{\tilde{D}}}^2 I_6(t)\right] \right\} . \end{aligned}$$

It seems plausible that these results extend to the case when there is no maximal degree but that would involve results for countably infinite matrices which we do not consider here.

Recall that the processes $\{{\hat{Z}}_i(t):t \ge 0\}$ $(i=1,2,\ldots ,K)$ are mutually independent. It follows using the central limit theorem that, for sufficiently large *K*, the process $\{Z(t):t \ge 0\}$, which approximates the prevalence of infection during the early growth of an epidemic, is approximately Gaussian with mean function given by (8.1) and covariance function given by (8.3).

## Numerical examples

### Forward simulations

**Fig. 2:**
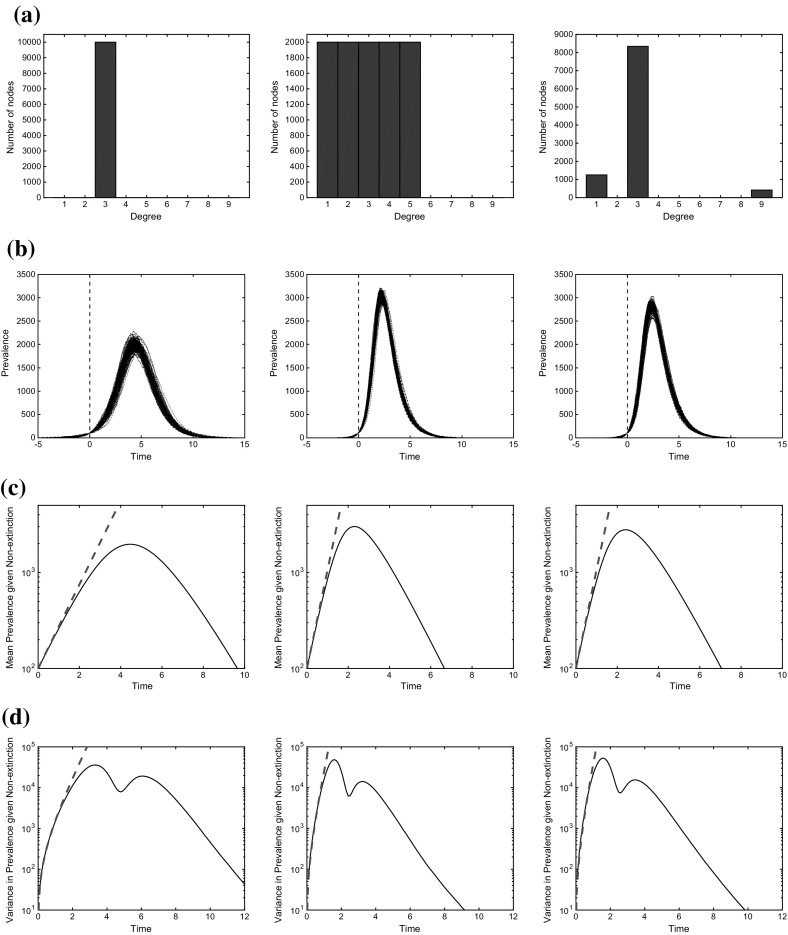
Epidemic simulations that set $\mathrm {time}=0$ when prevalence is equal to 100. Parameters are $\tau =2$, $\gamma =1$ throughout. **a** Degree distribution histograms, **b** 100 sample trajectories, **c** Mean prevalence. *Black solid* simulations; *Red dashed* branching process **d** Variance in prevalence. *Black solid* simulations; *Red dashed* branching process (colour figure online)

We conducted a series of numerical experiments to provide specific examples of the general results presented here. $M=10^4$ Monte Carlo simulations were performed on three different configuration model networks, each of size $N=10^4$, and with the degree distributions shown in Fig. 2 Row (a). (Note that each Monte Carlo simulation consisted of first simulating a network and then simulating a single epidemic on it.) Two different scenarios were considered. In the first – most commonly considered in the literature when simulations are compared to analytic approaches – $\mathrm {time}=0$ was defined as the first point when prevalence is at a given level, *K*. In our simulations we took $K=100$, but in general *K* should take a value where the probability of extinction has become negligible, but the depletion of the susceptible population has not had a significant effect on the epidemic dynamics. In the second, each epidemic was started from one node, picked uniformly at random, so the probability of extinction played a major role. This scenario is less commonly considered when comparing real-time simulated epidemics to differential equation models because the latter are typically designed to hold when the epidemic is already established.

Since analytic results for the probabilities of extinction $\pi _k(t)$ are not available, the branching process results required numerical integration of ordinary differential equations (in our case using Runge–Kutta methods). We stress that the computational effort required to do this is much less than that involved in performing Monte Carlo simulations, and has the benefit of not depending on *N*.

The results for the first approach (restarting time at the first time prevalence reaches 100) are given in Fig. 2. Row (b) shows sample trajectories (which all agree on prevalence at time 0). Row (c) shows the simulated mean after time 0 on a logarithmic scale, which initially grows at the constant rate predicted by the branching process model, and then reduces as the susceptible population is depleted. Row (d) shows the variance, which has not converged to its asymptotic growth regime by the time prevalence is equal to 100, an effect that is captured by the branching process model. The variance does not take its largest value at the peak prevalence, but instead has local maxima before and after the peak.

**Fig. 3:**
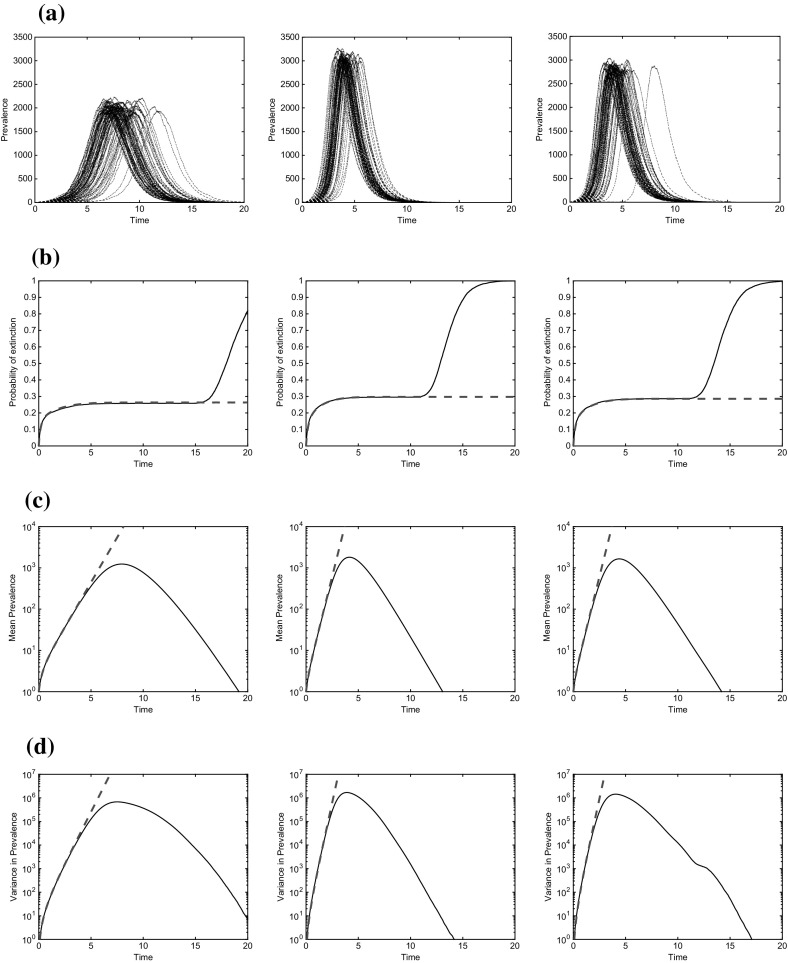
Epidemic simulations starting from one node selected uniformly at random. Parameters are $\tau =2$, $\gamma =1$ throughout. Degree distributions are as for Fig. 2 above. **a** 100 sample trajectories, **b** Extinction probabilities, *Black solid* simulations; *Red dashed* branching process, **c** Mean prevalence. *Black solid* simulations; *Red dashed* branching process, **d** Variance in prevalence. *Black solid* simulations; *Red dashed* branching process (colour figure online)

Figure 3 shows the results for the second approach in which there is one randomly chosen initial infective at time 0. Row (a) shows some sample trajectories. Row (b) shows the extinction probabilities, which are accurately captured in the branching process model until very close to the end of the epidemic when prevalence is low and extinction becomes likely again. Row (c) shows the mean, which does not start growing at a constant rate with the convergence rate accurately captured in the branching process model. Row (d) shows the variance and convergence onto its asymptotic value; in this case there is a single maximum just before the peak in prevalence.

Another important point is that while mean numbers infected are comparable between Figs. 2 and 3, the variability in the time for the epidemic to take off, as well as the contribution from extinct epidemics, makes the real-time variance in Fig. 3 orders of magnitude larger than in Fig. 2.

### Statistical inference

In order to demonstrate the potential use of the real-time effective degree branching process model for statistical inference, we carried out a simulation study. Here we simulated one epidemic that took off on a configuration model network of size $N=10^6$ with degree distribution $D^{(3)}$ as in the right-hand column of Fig. 2 ($d^{(3)}_1 = 1/8$, $d^{(3)}_3 = 5/6$, $d^{(3)}_9 = 1/24$) and true rates ${\tau }_0 = 2$, ${\gamma }_0 = 1$. Letting *I*(*t*) be the prevalence of infection in the network model, we set time $t=0$ when $I(t)=100$ for the first time and make 40 evenly-spaced observations (with gap $\delta t = 0.05$ between each) of *I*(*t*) up to $t_{\mathrm {end}}=2$.

We then define an approximate likelihood based on the methods of Ross et al. (2006), in which a Gaussian process approximation based on known first and second moments is used, which will be more accurate for larger *N*, larger *I*(0) and smaller $\delta t$. There should be, however, no *a priori* obstacle to fitting our model to data on smaller populations even with incomplete data, for example by using Markov chain Monte Carlo methods to perform multiple imputation as suggested by O’Neill and Roberts (1999).

Explicitly, we let the probability density function *f* for sequential observations be given by

9.1 $$\begin{aligned} f(I(t+\delta t)|I(t)) = \mathscr {N}(\mathbb {E}[Z(t + \delta t) | Z(t)=I(t)] , \text {var}(Z(t + \delta t) | Z(t)=I(t)) ,\nonumber \\ \end{aligned}$$

where $\mathscr {N}(m,V)$ is the probability density function of a normal distribution with mean *m* and variance *V*, and the expectation and variance of $Z(t+\delta t)|Z(t)=I(t)$ are given by the results of Sect. 8 above. The likelihood is then

9.2 $$\begin{aligned} L = \prod _{t\in \{0,\delta t, \ldots , t_{\mathrm {end}}-\delta t\}} f(I(t+\delta t)|I(t)) . \end{aligned}$$

We consider values of this likelihood across the range of rate constant parameters $\tau$ and $\gamma$ under two different degree distributions: the correct one, $D^{(3)}$, and a misspecified degree distribution $D^{(1)}$, which is the one used in the left-hand column of Fig. 2 ($d^{(1)}_3 = 1$).

**Fig. 4:**
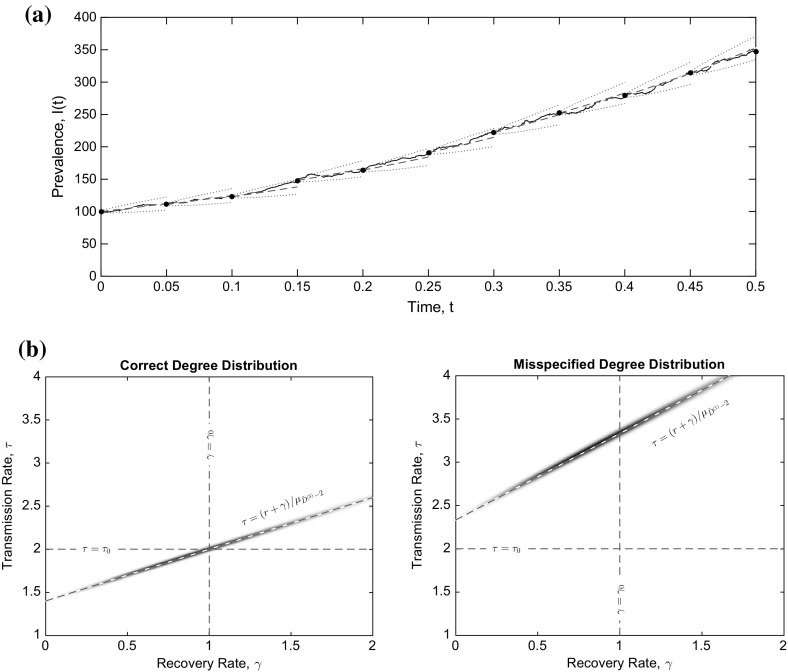
Simulation study. The *top plot* **a** shows the first quarter of the timepoints **a** Simulated epidemic, **b** Likelihood surfaces (observations as *black dots*, full trajectory as *black solid line*, Gaussian approximation mean as *red dashed line*, Gaussian approximation 95% prediction interval as *red dotted line*). The *bottom plots* **b** show likelihood surfaces for a Gaussian approximation model as described in Sect. 9.2. True parameters are ${\tau _0}=2$, ${\gamma _0}=1$. Correct degree distribution $D^{(3)}$ is as given in the third columns of Figs. 2 and 3 above, and the misspecified distribution $D^{(1)}$ is the distribution from the first column. Likelihood at a point is proportional to the intensity of shading, and with three straight lines, corresponding to the true values of $\tau , \gamma$ and *r*, are shown each plot as dashed red lines (color figure online)

Figure 4 shows the first quarter of the simulated epidemic together with the Gaussian process approximation, as well as likelihood surfaces for the correct and misspecified degree distributions. Performing maximum likelihood estimation using MATLAB’s mle() function allows us to obtain point estimates for parameters ${\hat{\tau }}$ and ${\hat{\gamma }}$, as well as asymptotic 95% confidence intervals and the parameter covariance matrix ${\hat{C}}$ from the inverse Hessian. We quote results to 2 significant figures; the asymptotic approximations also give very slightly negative lower confidence intervals for ${\hat{\gamma }}$ which we round up to 0. For the correct degree distribution we obtain

9.3 $$\begin{aligned} \begin{pmatrix} {{\hat{\tau }}}^{(3)}\\ {\hat{\gamma }}^{(3)} \end{pmatrix} = \begin{pmatrix} 1.9 &{}\quad [1.4,2.4]\\ 0.8 &{}\quad [0,1.7] \end{pmatrix} , \qquad \hat{\varvec{C}}^{(3)} = \begin{pmatrix} 0.069 &{}\quad 0.11\\ 0.11 &{}\quad 0.19 \end{pmatrix} , \end{aligned}$$

and for the misspecified degree distribution we obtain

9.4 $$\begin{aligned} \begin{pmatrix} {{\hat{\tau }}}^{(1)}\\ {\hat{\gamma }}^{(1)} \end{pmatrix} = \begin{pmatrix} 3.2 &{}\quad [2.3,4.0]\\ 0.8 &{}\quad [0,1.7] \end{pmatrix} , \qquad \hat{\varvec{C}}^{(1)} = \begin{pmatrix} 0.0045 &{}\quad 0.011\\ 0.011 &{}\quad 0.030 \end{pmatrix} . \end{aligned}$$

This shows that knowledge of the correct distribution allows both $\tau$ and $\gamma$ to be estimated; although as would be expected the early asymptotic growth rate *r* is much more closely constrained by simulated data than other directions in parameter space. It also shows that misspecification of the degree distribution allows *r* to be identified, but biases the estimate of, in this case, $\tau$.

## Concluding comments

### Summary of results

In this paper, we have provided explicit closed-form expressions for the real-time mean, variance and covariance function for disease prevalence during the early stages of the Markovian SIR model on a configuration model network, as well as deriving differential equations for the probabilities of extinction over time that are relatively numerically cheap to solve. These allow for a more explicit treatment of e.g. rates of convergence to asymptotic behaviour than has previously been possible.

### Future directions

We believe that the methods of real-time, multitype branching processes could be more widely applied in infectious disease epidemiology, since they provide results concerning extinction and variance that are not available using deterministic differential equation models. For example, the effective-degree based methodology presented here may be extended to include degree correlation (e.g. in the sense of Newman 2002) by keeping track of the actual, as well as effective, degrees of individuals, though the type space becomes larger and explicit analytic results are unlikely to be available. We note that there is increasing interest in the eradication of infections (e.g. Klepac et al. 2013) and that arguably calculating extinction probabilities and variability in outbreak sizes is of equal or greater importance in this context than calculation of mean behaviour.

The explicit closed-form expressions derived have the potential to enhance statistical work on epidemic prevalence curves. In particular, many empirically observed epidemics of human pathogens exhibit more variability around the trend than simple models would predict (see Black et al. 2014, particularly Section 1, for a discussion of this), which can bias parameter estimation if an insufficiently variable model is used. Application of our methods to real data would be an interesting extension of our work.

The possibility of a more general non-Markovian stochastic epidemic being approximated by an appropriate real-time branching process is raised by the results of Barbour and Reinert (2013) and it would be interesting to investigate whether our analysis could be adapted to this scenario.

Finally, there is the question of low-dimensional PGF-based modelling of the whole network epidemic that incorporates stochasticity accurately. For example, the work of Miller (2014) considered accounting for early fluctuations and Graham and House (2014) considered a diffusion approximation once early fluctuations were negligible, but the results presented here as well as those of Barbour and Reinert (2013) suggest that a more unified low-dimensional stochastic approach that explicitly models early fluctuations may be possible.

## Appendix 1: Convergence of moments

We determine sufficient conditions for the first two moments of the number of infectives in the epidemic $E_N$ among a population of *N* individuals to converge to the corresponding moments of the limiting branching process $\mathscr {B}$. For ease of exposition, in $E_N$ we assume that at time $t=0$ there is one infective and the remaining $N-1$ individuals are susceptible. The initial infective is chosen by sampling a stub uniformly at random from all stubs used to form the network, with the individual attached to that stub being the initial infective. The arguments are easily extended to other choices of initial infective(s).

In the independent and identically distributed (i.i.d.) degree case, we assume that a single sequence $D_1,D_2,\dots$ of i.i.d. copies of *D* is used to construct a sequence of epidemics $(E_N)$, where, for $N=1,2,\dots$, the epidemic $E_N$ is constructed using $D_1,D_2,\dots , D_N$. In the prescribed degree case (see Sect. 2.1), recall that $p_k^{(N)}$ $(k=0,1,\dots )$ denotes the empirical degree distribution in the epidemic $E_N$ and, for $f:\mathbb {R} \rightarrow \mathbb {R}$, let $\mu _{f\left( D^{(N)}\right) }=\sum _{k=0}^{\infty } p_k^{(N)} f(k)$.

For $N=1,2,\dots$ and $t \ge 0$, let $Y_N(t)$ be the number of infectives in $E_N$ at time *t* and let *Z*(*t*) be the number of individuals in the limiting branching process $\mathscr {B}$. Then arguing as in the proof of (Ball and Neal 2008, Theorem A.1), shows that in the i.i.d. degree case, if $\mu _D <\infty$ then the sequence of epidemics $(E_N)$ and the limiting branching process $\mathscr {B}$ can be constructed on a common probability space so that, with probability one, for any $t > 0$, $Y_N(u)$ and *Z*(*u*) coincide for all $u \in [0,t]$ for all sufficiently large *N*. The same conclusion holds in the prescribed degree case provided $p^{(N)}_k \rightarrow p_k$ $(k=0,1,\ldots )$ and $\mu _D^{(N)} \rightarrow \mu _D$ as $N \rightarrow \infty$, where $\sum _{k=0}^{\infty } p_k=1$ and $\mu _D < \infty$. Thus, under these conditions, in both cases, for any $t \ge 0$, $Y_N(t)$ converges almost surely to *Z*(*t*) as $N \rightarrow \infty$. We obtain further conditions, under which, for fixed $t \ge 0$, the sequence $\left( Y_N(t)^2\right)$ is uniformly integrable, which then (e.g. Grimmett and Stirzaker 2001, Chap. 7, Sect. 10) implies immediately that $\lim _{N \rightarrow \infty }\mathbb {E}\left[ Y_N(t)\right] = \mathbb {E}\left[ Z(t)\right] ,\lim _{N \rightarrow \infty }\mathbb {E}\left[ Y_N(t)^2\right] = \mathbb {E}\left[ Z(t)^2\right]$ and, for any $0 \le s \le t$, $\lim _{N \rightarrow \infty }\mathrm{cov}\left( Y_N(s),Y_N(t)\right) =\mathrm{cov}\left( Z(s), Z(t)\right)$. To show that $\left( Y_N(t)^2\right)$ is uniformly integrable it is sufficient to show that the sequence $\left( \mathbb {E}\left[ Y_N(t)^{2+\delta }\right] \right)$ is bounded above for some $\delta >0$.

For ease of exposition, we use notation from the i.i.d. degree case. The construction in Ball and Neal (2008) involves for each *N* constructing a realisation of a branching process, $\mathscr {B}_N$ say, which is defined analagously to $\mathscr {B}$ but using the empirical distribution of $D_1,D_2,\dots , D_N$ rather than the distribution of *D*. In $\mathscr {B}_N$, for each birth a stub is chosen independently and uniformly from all the $D_1+D_2+\dots +D_N$ stubs and the degree of the individual that the chosen stub belongs to gives the degree of the individual born at that birth. The process of infectives in $E_N$ follows $\mathscr {B}_N$ except when (i) a sampled stub has previously been chosen, in which case stubs are resampled until one that has not been chosen previously is obtained, or (ii) a sampled stub has not been chosen previously but is attached to an individual that has already been infected, in which case the corresponding birth and all descendants of that individual in $\mathscr {B}_N$ are ignored in $E_N$. Note that (i) implies that $\mathscr {B}_N$ need not be an almost sure upper bound for the process of infectives in $E_N$. The branching processes $\mathscr {B}_N$ $(N=1,2,\dots )$ and $\mathscr {B}$ are coupled so that with probability one, for any fixed $t>0$, $\mathscr {B}_N$ and $\mathscr {B}$ coincide over [0, *t*] for all sufficiently large *N*.

Let $D_{(1)},D_{(2)},\dots ,D_{(N)}$ be the order statistics of $D_1,D_2,\dots , D_N$, i.e. $D_1,D_2,\dots , D_N$ arranged in increasing order. For $\epsilon \in (0,1)$, let $\mathscr {B}_{N,\epsilon }$ be the branching process that is defined analagously to $\mathscr {B}$, but using the empirical distribution of $D_{([N\epsilon ]+1)},D_{([N\epsilon ]+2)},\dots , D_{(N)}$. (For $x \in \mathbb {R}$, [*x*] denotes the greatest integer $\le x$.) For $t \ge 0$, let $Z_ {N,\epsilon }(t)$ be the number of individuals alive in $\mathscr {B}_{N,\epsilon }$ at time *t* and let $T_{N,\epsilon }(t)$ denote the total progeny of $\mathscr {B}_{N,\epsilon }$ by time *t*, including the initial ancestor. Then $Y_N(t)\overset{st}{\le }Z_ {N,\epsilon }(t)$, provided that $T_{N,\epsilon }(t) \le N\epsilon$, where $\overset{st}{\le }$ denotes stochastically smaller than. (Up until $[N \epsilon ]$ infections have occurred in $E_N$, the empirical distribution of the degrees of unsampled stubs, where the degree of a stub is the degree of the individual to which it is attached, is stochastically smaller than that of the stubs belonging to the top $N-[N \epsilon ]$ individuals when ordered by degree.) As $Y_N(t)$ is at most *N*, it follows that

$$\begin{aligned} \mathbb {E}\left[ Y_N(t)^{2+\delta }\right] \le \mathbb {E}\left[ Z_ {N,\epsilon }(t)^{2+\delta }\right] +N^{2+\delta }\mathbb {P}\left( T_{N,\epsilon }(t)>N\epsilon \right) . \end{aligned}$$

By Markov’s inequality,

$$\begin{aligned} \mathbb {P}\left( T_{N,\epsilon }(t)>N\epsilon \right) \le \frac{1}{(N\epsilon )^{2+\delta }}\mathbb {E}\left[ T_{N,\epsilon }(t)^{2+\delta }\right] . \end{aligned}$$

Also, $$Z_ {N,\epsilon }(t) \overset{st}{\le } T_ {N,\epsilon }(t)$$, so

10.1 $$\begin{aligned} \mathbb {E}\left[ Y_N(t)^{2+\delta }\right] \le \left( 1+\epsilon ^{-(2+\delta )}\right) \mathbb {E}\left[ T_{N,\epsilon }(t)^{2+\delta }\right] . \end{aligned}$$

We now bound $\mathbb {E}\left[ T_{N,\epsilon }(t)^{2+\delta }\right]$, for $\delta \in (0,1)$. Note that this moment is smaller than the corresponding moment for the branching process in which $\gamma =0$ and individuals retain their original effective degree throughout their lifetime. Moreover, by rescaling the time axis, we can assume without loss of generality that $\tau =1$. Thus consider a multitype Markov birth process, with types $0,1,\dots ,J$, in which an individual of type *i* gives birth at rate *i* and the types of successive births are i.i.d. with probability mass function ${\hat{p}}_j$ $(j=0,1,\dots ,J)$. For $i=0,1,\dots ,J$, let ${\hat{T}}_i(t)$ denote the total number of individuals alive in this process at time *t* given that at time 0 there is one indivdiual, whose type is *i*. For $\alpha >0$ and $t \ge 0$, let ${\hat{\mu }}_i^{(\alpha )}(t)=\mathbb {E}\left[ {\hat{T}}_i(t)^{\alpha }\right]$ $(i=0,1,\dots ,J)$ and let ${\hat{\mu }}^{(\alpha )}(t)=\sum _{i=0}^J {\hat{p}}_i {\hat{\mu }}_i^{(\alpha )}(t)$. It is possible using a backward argument to derive explicit expressions for ${\hat{\mu }}_i^{(k)}(t)$ for $k=1,2,\dots$, though the algebra soon becomes very tedious. As our aim is to bound ${\hat{\mu }}^{(2+\delta )}(t)$, we simply derive bounds for ${\hat{\mu }}_i^{(1)}(t),{\hat{\mu }}_i^{(2)}(t)$ $(i=0,1,\dots ,J)$ and finally ${\hat{\mu }}^{(2+\delta )}(t)$. Moreover, our bounds are deliberately coarse to facilitate easy application to $\mathbb {E}\left[ T_{N,\epsilon }(t)^{2+\delta }\right]$. For $f:\mathbb {R} \rightarrow \mathbb {R}$, let ${\hat{\mu }}_{f(D)}=\sum _{i=0}^J f(i) {\hat{p}}_i$.

For $i=0,1,\dots ,J$, the backward equation for ${\hat{\mu }}_i^{(1)}(t)$ is

10.2 $$\begin{aligned} \frac{\mathrm {d}{\hat{\mu }}_i^{(1)}}{\mathrm {d}t}=i{\hat{\mu }}^{(1)}, \qquad {\hat{\mu }}_i^{(1)}(0)=1. \end{aligned}$$

Multiplying (10.2) by ${\hat{p}}_i$ and summing over $i=0,1,\dots ,J$, yields

$$\begin{aligned} \frac{\mathrm {d}{\hat{\mu }}^{(1)}}{\mathrm {d}t}={\hat{\mu }}_D{\hat{\mu }}^{(1)}, \qquad {\hat{\mu }}^{(1)}(0)=1. \end{aligned}$$

Thus

10.3 $$\begin{aligned} {\hat{\mu }}^{(1)}(t)=\mathrm{e}^{{\hat{\mu }}_D t}, \end{aligned}$$

which on substituting into (10.2) yields ${\hat{\mu }}_i^{(1)}=1+i\int _0^t \mathrm{e}^{{\hat{\mu }}_D s} \mathrm {d}s$, so

10.4 $$\begin{aligned} {\hat{\mu }}_i^{(1)}\le i(1+t)\mathrm{e}^{{\hat{\mu }}_D t}\qquad (i=1,2,\dots ,J). \end{aligned}$$

For $i=0,1,\dots ,J$, the backward equation for ${\hat{\mu }}_i^{(2)}(t)$ is

10.5 $$\begin{aligned} \frac{\mathrm {d}{\hat{\mu }}_i^{(2)}}{\mathrm {d}t}=2i{\hat{\mu }}_i^{(1)}{\hat{\mu }}^{(1)}+i{\hat{\mu }}^{(2)}, \qquad {\hat{\mu }}_i^{(2)}(0)=1. \end{aligned}$$

Substituting from (10.3) and (10.4), and then multiplying (10.5) by ${\hat{p}}_i$ and summing over $i=0,1,\dots ,J$, yields

$$\begin{aligned} \frac{\mathrm {d}{\hat{\mu }}^{(2)}}{\mathrm {d}t}\le 2{\hat{\mu }}_{D^2}(1+t)\mathrm{e}^{2{\hat{\mu }}_D t} +{\hat{\mu }}_D {\hat{\mu }}^{(2)}, \qquad {\hat{\mu }}^{(2)}(0)=1, \end{aligned}$$

whence

$$\begin{aligned} {\hat{\mu }}^{(2)}(t)\le & {} \mathrm{e}^{{\hat{\mu }}_D t}+\int _0^t \mathrm{e}^{{\hat{\mu }}_D (t-s)}2{\hat{\mu }}_{D^2}(1+s)\mathrm{e}^{2{\hat{\mu }}_D s}\mathrm {d}s\\\le & {} \left( 1+2{\hat{\mu }}_{D^2} t(1+t)\right) \mathrm{e}^{2{\hat{\mu }}_D t}. \end{aligned}$$

Substituting this bound into (10.5) and noting that the right-hand side of (10.5) is increasing in *t* yields

10.6 $$\begin{aligned} {\hat{\mu }}_i^{(2)}(t) \le i^2 g(t,{\hat{\mu }}_{D^2})\mathrm{e}^{2{\hat{\mu }}_D t} \qquad (i=1,2,\dots ,J), \end{aligned}$$

where

10.7 $$\begin{aligned} g(t,{\hat{\mu }}_{D^2})=1+t\left[ 1+2(1+t)(1+{\hat{\mu }}_{D^2} t)\right] . \end{aligned}$$

Let $\delta \in (0,1)$. For $i=0,1,\dots ,J$, the backward equation for ${\hat{\mu }}_i^{(2+\delta )}(t)$ is

10.8 $$\begin{aligned} \frac{\mathrm {d}{\hat{\mu }}_i^{(2+\delta )}}{\mathrm {d}t}=-i {\hat{\mu }}_i^{(2+\delta )}(t)+i\mathbb {E}\left[ \left( {\hat{T}}_i(t)+{\hat{T}}(t)\right) ^{2+\delta }\right] , \quad {\hat{\mu }}_i^{(2+\delta )}(0)=1,\qquad \end{aligned}$$

where ${\hat{T}}_i(t)$ and ${\hat{T}}(t)$ are independent and ${\hat{T}}(t)$ is distributed as a mixture of ${\hat{T}}_0(t), {\hat{T}}_1(t),\dots ,{\hat{T}}_J(t)$ with mixing probabilities ${\hat{p}}_0, {\hat{p}}_1,\dots , {\hat{p}}_J$.

Let *a* and *b* be nonnegative real numbers. Application of the mean value theorem yields that

$$\begin{aligned} (a+b)^{2+\delta } \le a^{2+\delta }+(2+\delta )b(a+b)^{1+\delta }. \end{aligned}$$

Further, $$(a+b)^{1+\delta } \le 2^{\delta }\left( a^{1+\delta }+b^{1+\delta }\right) $$, so

$$\begin{aligned} (a+b)^{2+\delta } \le a^{2+\delta }+2^{\delta }(2+\delta )\left( b a^{1+\delta }+b^{2+\delta }\right) . \end{aligned}$$

Setting $a={\hat{T}}_i(t)$ and $b={\hat{T}}(t)$ in this inequality, taking expectations exploiting the independence of ${\hat{T}}_i(t)$ and ${\hat{T}}(t)$, substituting into (10.8) and noting that $2^{\delta }(2+\delta ) <6$ gives

10.9 $$\begin{aligned} \frac{\mathrm {d}{\hat{\mu }}_i^{(2+\delta )}}{\mathrm {d}t}\le 6i\left[ {\hat{\mu }}^{(1)}(t){\hat{\mu }}_i^{(1+\delta )}(t) +{\hat{\mu }}^{(2+\delta )}(t)\right] , \qquad {\hat{\mu }}_i^{(2+\delta )}(0)=1. \end{aligned}$$

Now

$$\begin{aligned} {\hat{\mu }}_i^{(2)}(t)=\mathbb {E}\left[ \left( {\hat{T}}_i(t)^{1+\delta }\right) ^{\frac{2}{1+\delta }}\right] \ge \left( {\hat{\mu }}_i^{(1+\delta )}(t)\right) ^{\frac{2}{1+\delta }}, \end{aligned}$$

by Jensen’s inequality, so

10.10 $$\begin{aligned} {\hat{\mu }}_i^{(1+\delta )}(t)\le \left( {\hat{\mu }}_i^{(2)}(t)\right) ^{\frac{1+\delta }{2}} \le i^{1+\delta }g(t,{\hat{\mu }}_{D^2})\mathrm{e}^{(1+\delta ){\hat{\mu }}_D t}, \end{aligned}$$

using (10.6) and noting that $g(t,{\hat{\mu }}_{D^2})\ge 1$. Substituting (10.10) into (10.9), multiplying by ${\hat{p}}_i$ and summing over $i=0,1,\dots ,J$, yields

$$\begin{aligned} \frac{\mathrm {d}{\hat{\mu }}^{(2+\delta )}}{\mathrm {d}t}\le 6 {\hat{\mu }}_{D^{2+\delta }}g(t,{\hat{\mu }}_{D^2})\mathrm{e}^{(2+\delta ){\hat{\mu }}_D t}+6 {\hat{\mu }}_D {\hat{\mu }}^{(2+\delta )}. \end{aligned}$$

Hence,

10.11 $$\begin{aligned} {\hat{\mu }}^{(2+\delta )}\le & {} \mathrm{e}^{6{\hat{\mu }}_D t}+\int _0^t \mathrm{e}^{6{\hat{\mu }}_D (t-s)} 6 {\hat{\mu }}_{D^{2+\delta }}g(s,{\hat{\mu }}_{D^2})\mathrm{e}^{(2+\delta ){\hat{\mu }}_D s} \mathrm {d}s\nonumber \\\le & {} \mathrm{e}^{6{\hat{\mu }}_D t}\left[ 1+6 {\hat{\mu }}_{D^{2+\delta }}tg(t,{\hat{\mu }}_{D^2})\right] . \end{aligned}$$

We return to epidemics on networks and introduce some more notation. For $\epsilon \in (0,1)$, let $k_0(\epsilon )=\min \{k:p_0+p_1+\dots ,p_k > \epsilon \}$ and, for $k=0,1,\dots$, let

$$\begin{aligned} p_k(\epsilon )= {\left\{ \begin{array}{ll} 0 &{} \text { if } k<k_0(\epsilon ) ,\\ 1-\frac{1}{1-\epsilon }\sum _{k=k_0(\epsilon )}^{\infty } p_k &{} \text { if } k= k_0(\epsilon ) ,\\ \frac{p_k}{1-\epsilon } &{} \text { if } k> k_0(\epsilon ) . \end{array}\right. } \end{aligned}$$

For $f:\mathbb {R} \rightarrow \mathbb {R}$, let $\mu _{f(D(\epsilon ))}=\sum _{k=0}^\infty p_k(\epsilon ) f(k)$. As above, we assume without loss of generality that $\tau =1$.

Consider first the model with prescribed degrees. Let $D_{(1)}^{(N)},D_{(2)}^{(N)},\dots ,D_{(N)}^{(N)}$ be the order statistics of $D_1^{(N)},D_2^{(N)},\dots , D_N^{(N)}$. For $\epsilon \in (0,1)$, let $p_k^{(N)}(\epsilon )=(N-[N\epsilon ])^{-1}\sum _{i=N-[N\epsilon ]}^N \delta _{k,D_{(i)}^{(N)}}$ $(k=0,1,\dots )$ and, for $f:\mathbb {R} \rightarrow \mathbb {R}$, let

$$\begin{aligned} \mu _{f(D^{(N)}(\epsilon ))}=\sum _{k=0}^\infty p_k^{(N)}(\epsilon ) f(k)=\frac{1}{N-[N\epsilon ]}\sum _{k=[N\epsilon ]+1}^N f\left( D_{(k)}^{(N)}\right) ^\alpha . \end{aligned}$$

Let ${\tilde{p}}^{(N)}_k(\epsilon )=kp_k^{(N)}(\epsilon )/\mu ^{(N)}_{D(\epsilon )}$ $(k=1,2,\dots )$. For $f:\mathbb {R} \rightarrow \mathbb {R}$, let

$$\begin{aligned} \mu _{f\left( {\tilde{D}}^{(N)}(\epsilon )\right) }=\sum _{k=1}^\infty {\tilde{p}}_k^{(N)}(\epsilon ) f(k)=\frac{\mu _{D^{(N)}(\epsilon )f\left( D^{(N)}(\epsilon )\right) }}{\mu _{D^{(N)}(\epsilon )}}. \end{aligned}$$

Fix the population size *N* and $\epsilon \in (0,1)$. In the birth process used to bound the right-hand side of (10.1), the types of individuals born are distributed according to ${\hat{p}}_i={\tilde{p}}^{(N)}_{i+1}(\epsilon )$ $(i=0,1,\dots ,J)$, where $J=\max \left\{ D_{(k)}^{(N)}:k=1,2,\dots ,N\right\}$. Thus, for $\delta \in (0,1)$, it follows using (10.1) and (10.11) that

$$\begin{aligned} \mathbb {E}\left[ Y_N(t)^{2+\delta }\right] \le h_1(N,\epsilon ,t), \end{aligned}$$

where

$$\begin{aligned} h_1(N,\epsilon ,t)= & {} \left( 1+\epsilon ^{-(2+\delta )}\right) \left[ 1+6\mu _{({\tilde{D}}^{(N)}(\epsilon )-1)^{2+\delta }}t g\left( t,\mu _{({\tilde{D}}^{(N)}(\epsilon )-1)^2}\right) \exp \left( 6\mu _{{\tilde{D}}^{(N)}(\epsilon )-1}t\right) \right] . \end{aligned}$$

Suppose that there exists $\delta \in (0,1)$ such that $\mu _{D^{3+\delta }}<\infty$ and $\mu _{(D^{(N)})^{3+\delta }} \rightarrow \mu _{D^{3+\delta }}$ as $N \rightarrow \infty$. It is easily verified that these conditions imply that, for each $\epsilon \in (0,1)$ and any $\alpha \in [0,3+\delta ]$, $\mu _{D(\epsilon )^{\alpha }}<\infty$ and $\mu _{(D^{(N)}(\epsilon ))^\alpha } \rightarrow \mu _{D(\epsilon )^{\alpha }}$ as $N \rightarrow \infty$. Hence, for any $\alpha \in [0,2+\delta ]$, $\mu _{({\tilde{D}}^{(N)}(\epsilon )-1)^\alpha } \rightarrow \mu _{({\tilde{D}}(\epsilon )-1)^\alpha }$ as $N \rightarrow \infty$, where $\mu _{({\tilde{D}}(\epsilon )-1)^\alpha }< \infty$. It follows that $h_1(N,\epsilon ,t) \rightarrow h_1(\epsilon ,t)$ as $N \rightarrow \infty$, where

$$\begin{aligned} h_1(\epsilon ,t)= & {} \left( 1+\epsilon ^{-(2+\delta )}\right) \left[ 1+6\mu _{({\tilde{D}}(\epsilon )-1)^{2+\delta }}t g\left( t,\mu _{({\tilde{D}}(\epsilon )-1)^2}\right) \exp \left( 6 \mu _{{\tilde{D}}(\epsilon )-1}t\right) \right] <\infty . \end{aligned}$$

Thus the sequence $\left( \mathbb {E}\left[ Y_N(t)^{2+\delta }\right] \right)$ is bounded and, for any $t \ge 0$ and any $\alpha \in [0,2]$, $\mathbb {E}\left[ Y_N(t)^\alpha \right] \rightarrow \mathbb {E}\left[ Z(t)^\alpha \right]$ as $N \rightarrow \infty$.

Turn now to the model with i.i.d. degrees. Recall that we construct a sequence of epidemics $(E_N)$ from a single sequence $D_1,D_2,\dots$ of i.i.d. copies of *D*. For $N=1,2,\dots$, let $D^{(N)}_k=D_k$ ($k=1,2,\dots ,N$). Using the formulae derived previously for the prescribed degree case, but noting that now the degrees are random, by conditioning on the degree sequence $D_1,D_2,\dots$ we obtain that

10.12 $$\begin{aligned} \mathbb {E}\left[ Y_N(t)^{2+\delta }\right] \le h_2(N,\varepsilon ,t), \end{aligned}$$

where

$$\begin{aligned} h_2(N,\varepsilon ,t)=\mathbb {E}\left[ \left( 1+\varepsilon ^{-(2+\delta )}\right) \left\{ 1+6\mu _{(\tilde{D}^{(N)}(\varepsilon )-1)^{2+\delta }}t g\left( t,\mu _{(\tilde{D}^{(N)}(\varepsilon )-1)^2}\right) \exp \left( 6 \mu _{\tilde{D}^{(N)}(\varepsilon )-1}t\right) \right\} \right] . \end{aligned}$$

Fix $\varepsilon \in (0,1)$. Recalling the definition (10.7) of the function *g*, to obtain an upper bound for $h(N,\varepsilon ,t)$, it is sufficient to obtain upper bounds for

$$\begin{aligned}&\mathbb {E}\left[ \mu _{({\tilde{D}}^{(N)}(\varepsilon )-1)^{2+\delta }}\exp \left( 6 \mu _{{\tilde{D}}^{(N)}(\varepsilon )-1}t\right) \right] \qquad \text{ and }\\&\qquad \mathbb {E}\left[ \mu _{({\tilde{D}}^{(N)}(\varepsilon )-1)^{2+\delta }}\mu _{({\tilde{D}}^{(N)}(\varepsilon )-1)^{2}}\exp \left( 6 \mu _{{\tilde{D}}^{(N)}(\varepsilon )-1}t\right) \right] . \end{aligned}$$

Now $\mu _{({\tilde{D}}^{(N)}(\varepsilon )-1)^{2}} \le \mu _{({\tilde{D}}^{(N)}(\varepsilon )-1)^{2+\delta }}$ and, by Jensen’s inequality, $\left( \mu _{({\tilde{D}}^{(N)}(\varepsilon )-1)^{2+\delta }}\right) ^2 \le \mu _{({\tilde{D}}^{(N)}(\varepsilon )-1)^{2(2+\delta )}}$, so, since $\delta \in (0,1)$, it is sufficient to obtain an upper bound for $\mathbb {E}\left[ \mu _{({\tilde{D}}^{(N)}(\varepsilon )-1)^6}\exp \left( 6 \mu _{{\tilde{D}}^{(N)}(\varepsilon )-1}t\right) \right]$. Further, using the Cauchy-Schwarz inequality,

$$\begin{aligned} \mathbb {E}\left[ \mu _{({\tilde{D}}^{(N)}(\varepsilon )-1)^6}\exp \left( 6 \mu _{{\tilde{D}}^{(N)}(\varepsilon )-1}t\right) \right] \le \sqrt{ \mathbb {E}\left[ \mu _{({\tilde{D}}^{(N)}(\varepsilon )-1)^6}^2\right] \mathbb {E}\left[ \exp \left( 12 \mu _{{\tilde{D}}^{(N)}(\varepsilon )-1}t\right) \right] }. \end{aligned}$$

Let $M_{D^2}(\theta )=\mathbb {E}\left[ \exp \left( \theta D^2\right) \right]$ ($\theta \in \mathbb {R}$) be the moment-generating function of $D^2$ and suppose that there exists $\theta _0>0$ such that $M_{D^2}(\theta _0)< \infty$. Note that this implies that $\mathbb {E}\left[ D^\alpha \right] <\infty$ for all $\alpha \ge 0$.

Assume first that $p_0=0$, so $\mu _{D^{(N)}(\varepsilon )}\ge 1$ almost surely. Now

10.13 $$\begin{aligned} \mu _{({\tilde{D}}^{(N)}(\varepsilon )-1)^6}= & {} \frac{\mu _{D^{(N)}(\varepsilon )\left( D^{(N)}(\varepsilon )-1)^6\right) }}{\mu _{D^{(N)}(\varepsilon )}}\nonumber \\\le & {} \mu _{D^{(N)}(\varepsilon )\left( D^{(N)}(\varepsilon )-1)^6\right) }\qquad \text{ almost } \text{ surely }\nonumber \\= & {} \frac{1}{N-[N\varepsilon ]}\sum _{k=[N\varepsilon ]+1}^N D^{(N)}_{(k)}\left( D^{(N)}_{(k)}-1\right) ^6\nonumber \\\le & {} \frac{1}{N(1-\varepsilon )}\sum _{k=1}^N D_k^7, \end{aligned}$$

since $D^{(N)}_k=D_k$ ($k=1,2,\dots ,N$). Thus, since $D_1,D_2,\dots ,D_N$ are i.i.d.,

$$\begin{aligned} \mathbb {E}\left[ \mu _{({\tilde{D}}^{(N)}(\varepsilon )-1)^6}^2\right]\le & {} \frac{1}{N^2(1-\varepsilon )^2}\mathbb {E}\left[ \left( \sum _{k=1}^N D_k^7\right) ^2\right] \\= & {} \frac{1}{N^2(1-\varepsilon )^2}\left[ N\mu _{D^{14}}+N(N-1)\mu _{D^7}^2\right] \\\le & {} \frac{1}{(1-\varepsilon )^2}\left( \mu _{D^{14}}+\mu _{D^7}^2\right) . \end{aligned}$$

A similar argument to (10.13) yields $\mu _{({\tilde{D}}^{(N)}(\varepsilon )-1)}\le \frac{1}{N(1-\varepsilon )}\sum _{k=1}^N D_k^2$, so

$$\begin{aligned} \mathbb {E}\left[ \exp \left( 12 \mu _{{\tilde{D}}^{(N)}(\varepsilon )-1}t\right) \right] \le \mathbb {E}\left[ \exp \left( \frac{12t}{N(1-\varepsilon )}\sum _{k=1}^N D_k^2 \right) \right] = \left[ M_{D^2}\left( \frac{12t}{N(1-\varepsilon )}\right) \right] ^N. \end{aligned}$$

Fix $t \ge 0$. Then $\mathbb {E}\left[ \exp \left( 12 \mu _{{\tilde{D}}^{(N)}(\varepsilon )-1}t\right) \right] <\infty$ for $N \ge N(\varepsilon ,t)$, where $N(\varepsilon ,t)=\frac{12t}{(1-\varepsilon )\theta _0}$. Now $M_{D^2}(\theta )=1+\mu _{D^2}\theta +o(\theta )$ as $\theta \rightarrow 0$, so

$$\begin{aligned} \lim _{N \rightarrow \infty } \mathbb {E}\left[ \exp \left( 12 \mu _{{\tilde{D}}^{(N)}(\varepsilon )-1}t\right) \right]\le & {} \lim _{N \rightarrow \infty }\left[ 1+\frac{12t}{N(1-\varepsilon )}\mu _{D^2}+o\left( \frac{1}{N}\right) \right] ^N\\= & {} \exp \left( \frac{12t\mu _{D^2}}{1-\varepsilon }\right) <\infty . \end{aligned}$$

The above arguments show that there exists $h_2(\varepsilon ,t)<\infty$ such that $h_2(N,\varepsilon ,t)<h_2(\varepsilon ,t)$ for all $N \ge N(\varepsilon ,t)$. Now $Y_N(t) \le N$ for all *N*, so $\mathbb {E}\left[ Y_N(t)^{2+\delta }\right] \le N(\varepsilon ,t)^{2+\delta }$ for $N<N(\varepsilon ,t)$. Thus, recalling (10.12), the sequence $\left( \mathbb {E}\left[ Y_N(t)^{2+\delta }\right] \right)$ is bounded and, for any $\alpha \in [0,2]$, $\mathbb {E}\left[ Y_N(t)^\alpha \right] \rightarrow \mathbb {E}\left[ Z(t)^\alpha \right]$ as $N \rightarrow \infty$.

Suppose now that $p_0>0$. Then $D \overset{st}{\le } D'$, where $D'$ has distribution given by $\mathbb {P}(D'=1)=p_0+p_1$ and $\mathbb {P}(D'=k)=p_k$ ($k=2,3,\dots$). It follows that for fixed population size *N*, fixed $\varepsilon \in (0,1)$ and any $t \ge 0$, $T_{N,\varepsilon }(t) \overset{st}{\le } T_{N,\varepsilon }'(t)$, where $T_{N,\varepsilon }'(t)$ is the total progeny at time *t* of the branching process defined analagously to $\mathscr {B}_{N, \varepsilon }$ but using the empirical distribution of $D_1',D_2',\dots ,D_N'$, where $D_1',D_2',\dots ,D_N'$ are i.i.d. copies of $D'$. Further, $M_{D'^2}(\theta _0)<\infty$ if $M_{D^2}(\theta _0)<\infty$ and the above argument can be used to show that the sequence $\left( \mathbb {E}\left[ Y_N(t)^{2+\delta }\right] \right)$ is bounded.

The above argument is easily adapted to show that in the prescribed degree case $\lim _{N \rightarrow \infty }\mathbb {E}\left[ Y_N(t)\right] = \mathbb {E}\left[ Z(t)\right]$ under the weaker condition that there exists $\delta >0$ such that $\mu _{D^{2+\delta }}<\infty$ and $\mu _{(D^{(N)})^{2+\delta }} \rightarrow \mu _{D^{2+\delta }}$ as $N \rightarrow \infty$. Moreover, although we have not worked through all of the details, it seems likely that the argument can also be adapted to prove that, for any $\alpha >1$, if there exists $\delta >1$ such that $\mu _{D^{\alpha +\delta }}<\infty$ and $\mu _{(D^{(N)})^{\alpha +\delta }} \rightarrow \mu _{D^{\alpha +\delta }}$ as $N \rightarrow \infty$ then $\lim _{N \rightarrow \infty }\mathbb {E}\left[ Y_N(t)^\alpha \right] = \mathbb {E}\left[ Z(t)^\alpha \right]$ for all $t \ge 0$. Again we have not worked through all of the details but in the i.i.d. degree case it seems likely that the above condition on $M_{D^2}(\theta )$ quarantees that $\lim _{N \rightarrow \infty }\mathbb {E}\left[ Y_N(t)^\alpha \right] = \mathbb {E}\left[ Z(t)^\alpha \right]$ for all $\alpha ,t \ge 0$. Finally, in the i.i.d. degree case it seems likely that weaker conditions will suffice when the limiting branching process $\mathscr {B}$ is subcritical, i.e. when $r<0$, as in that case the exponential functions appearing in $\mathbb {E}\left[ T_{N,\varepsilon }(t)^{2+\delta }\right]$, prior to taking expectations, will all have negative arguments.

## Appendix 2: Eigenvalues of $\varvec{{\varOmega }}$

Let $\varvec{A} =[a_{l,k}]= \varvec{{\varOmega }} - \lambda \varvec{I}$. Observe that $a_{0,0}=-(\lambda + \gamma )$, $a_{0,k}=0$ for $k=1,2,\ldots ,k_{\mathrm {max}}$, $a_{l,k_{\mathrm {max}}}=0$ for $l=1,2,\ldots ,k_{\mathrm {max}}-1$ and $a_{k_{\mathrm {max}},k_{\mathrm {max}}}=-(k_{\mathrm {max}}\tau + \lambda + \gamma )$. Thus, expanding the determinant $| \varvec{A} |$ along the 0th row and then the cofactor $A_{0,0}$ down the last column yields

10.14 $$\begin{aligned} | \varvec{A} |=(\lambda + \gamma )(k_{\mathrm {max}}\tau + \lambda + \gamma )| \varvec{B} | , \end{aligned}$$

where

$$\begin{aligned} \varvec{B}&= \begin{bmatrix} \tau ({\tilde{p}}_2 -1) -\lambda - \gamma&\quad \tau {\tilde{p}}_3&\quad \cdots&\quad \tau {\tilde{p}}_{k_{\mathrm {max}}} \\ 2\tau ({\tilde{p}}_2 + 1)&\quad 2\tau ({\tilde{p}}_3 -1) -\lambda - \gamma&\quad \cdots&\quad 2 \tau {\tilde{p}}_{k_{\mathrm {max}}} \\ \vdots&\quad \vdots&\quad \ddots&\quad \vdots \\ (k_{\mathrm {max}}-1)\tau {\tilde{p}}_2&\quad (k_{\mathrm {max}}-1) \tau {\tilde{p}}_3&\quad \cdots&\quad (k_{\mathrm {max}}-1) \tau ({\tilde{p}}_{k_{\mathrm {max}}}-1) - \lambda - \gamma \end{bmatrix}.\\ \end{aligned}$$

More precisely, $\varvec{B}$ is the $(k_{\mathrm {max}}-1) \times (k_{\mathrm {max}}-1)$ matrix with elements

$$\begin{aligned} b_{l,k}= \tau l \left( {\tilde{p}}_{k+1} + \delta _{l,k+1}\right) -(\gamma + \tau l + \lambda ) \delta _{l,k} \qquad (l,k=1,2,\ldots ,k_{\mathrm {max}}-1) . \end{aligned}$$

Subtracting $l \times$ the first row of $\varvec{B}$ from the *l*th row of $\varvec{B}$, for $l=2,3,\ldots ,k_{\mathrm {max}}-1$, now gives $| \varvec{B} |=| \varvec{C} |$, where

$$\begin{aligned} \varvec{C}&= \begin{bmatrix} \tau ({\tilde{p}}_2 -1) -\lambda - \gamma&\quad \tau {\tilde{p}}_3&\quad \cdots&\quad \tau {\tilde{p}}_{k_{\mathrm {max}}} \\ 2(2\tau + \lambda + \gamma )&\quad -2\tau -\lambda - \gamma&\quad \cdots&\quad 0 \\ 3(\tau + \lambda + \gamma )&\quad 3\tau&\quad \cdots&\quad 0 \\ \vdots&\quad \vdots&\quad \ddots&\quad \vdots \\ (k_{\mathrm {max}}-1) (\tau + \lambda + \gamma )&\quad 0&\quad \cdots&\quad -(k_{\mathrm {max}}-1) \tau - \lambda - \gamma \end{bmatrix} \end{aligned}$$

has elements given by

$$\begin{aligned} c_{1,k}&=\tau {\tilde{p}}_{k+1} -(\gamma + \tau + \lambda ) \delta _{1,k}\qquad (k=1,2,\ldots ,k_{\mathrm {max}}-1), \\ c_{l,k}&= \tau l \delta _{l,k+1} -(\gamma + \tau l + \lambda ) \delta _{l,k} +l(\gamma + \tau + \lambda )\delta _{1,k}\\&(l=2,3,\ldots ,k_{\mathrm {max}}-1; k=1,2,\ldots ,k_{\mathrm {max}}-1) . \end{aligned}$$

In particular, $c_{1,1}= \tau ({\tilde{p}}_2-1)-\gamma -\lambda$, $c_{1,k}=\tau {\tilde{p}}_{k+1}$ for $k=2,3,\ldots ,k_{\mathrm {max}}-1$, and $c_{l,k}=0$ for $2 \le l<k\le k_{\mathrm {max}}-1$. Thus, adding $k \times$ the *k*th column of $\varvec{C}$ to the first column of $\varvec{C}$, for $k=2,3,\ldots ,k_{\mathrm {max}}-1$, yields $| \varvec{C} |=| \varvec{D} |$, where

$$\begin{aligned} \varvec{D}&= \begin{bmatrix} \tau \left( \left( {\textstyle \sum _{l=0}^{k_{\mathrm {max}}}} l {\tilde{p}}_{l+1}\right) -1\right) -\lambda - \gamma&\quad \tau {\tilde{p}}_3&\quad \cdots&\quad \tau {\tilde{p}}_{k_{\mathrm {max}}} \\ 0&\quad -2\tau -\lambda - \gamma&\quad \cdots&\quad 0 \\ 0&\quad 3\tau&\quad \cdots&\quad 0 \\ \vdots&\quad \vdots&\quad \ddots&\quad \vdots \\ 0&\quad 0&\quad \cdots&\quad -(k_{\mathrm {max}}-1) \tau - \lambda - \gamma \end{bmatrix} . \end{aligned}$$

Note that $d_{1,1}=\tau \left[ \left( \sum _{l=0}^{k_{\mathrm {max}}} l {\tilde{p}}_{l+1}\right) -1\right] -\gamma -\lambda$ and, for $l \ge 2$, that $d_{l,1}=0, d_{l,l}=-(\gamma + \tau + \lambda )$ and $d_{l,k}=0$ for $k>l$. Thus expanding $| \varvec{D} |$ down the first column gives

$$\begin{aligned} | \varvec{D} |=\left\{ \tau \left[ \left( \sum _{l=0}^{k_{\mathrm {max}}} l {\tilde{p}}_{l+1}\right) -1\right] -\gamma -\lambda \right\} \left( -1\right) ^{k_{\mathrm {max}}-2}\prod _{l=2}^{k_{\mathrm {max}}-1} \left( l\tau +\gamma +\lambda \right) . \end{aligned}$$

Recalling (10.14) and $| \varvec{B} |=| \varvec{C} |=| \varvec{D} |$, it follows that the eigenvalues of $\varvec{{\varOmega }}$ are given by (3.3).

## Appendix 3: Derivation of variance

Recall the definition of $\varvec{C} _k$ at (4.2). Note, using (2.4), that for $k\in \mathscr {K}$,

10.15 $$\begin{aligned} \mathbf {1} ^{\!\top } \varvec{C} _k \mathbf {1}&= \tau k + \gamma ,\nonumber \\ \mathbf {1} ^{\!\top } \varvec{C} _k \mathbf {n}&= \mathbf {n} ^{\!\top } \varvec{C} _k \mathbf {1} = (r + 2\gamma ) k ,\nonumber \\ \mathbf {n} ^{\!\top } \varvec{C} _k \mathbf {n}&= \tau \mu _{({\tilde{D}}-2)^2} k + \gamma k^2 . \end{aligned}$$

Now let $\mathbf {c} _{11}$ be a column vector whose *k*th element is $\mathbf {1} ^{\!\top } \varvec{C} _k \mathbf {1}$, and define $\mathbf {c} _{1n}$ and $\mathbf {c} _{nn}$ similarly, using $\mathbf {1} ^{\!\top } \varvec{C} _k \mathbf {n}$ and $\mathbf {n} ^{\!\top } \varvec{C} _k \mathbf {n}$, respectively. Noting that $r + 2\gamma =\gamma +\tau \mu _{{\tilde{D}}-2}$, in this more compact notation (10.15) becomes

10.16 $$\begin{aligned} \mathbf {c} _{11}&= \tau \mathbf {n} + \gamma \mathbf {1},\nonumber \\ \mathbf {c} _{1n}&= \left( \gamma +\tau \mu _{{\tilde{D}}-2}\right) \mathbf {n} ,\nonumber \\ \mathbf {c} _{nn}&= \tau \mu _{({\tilde{D}}-2)^2} \mathbf {n} + \gamma \mathbf {n} _2 , \end{aligned}$$

and (4.3) yields

10.17 $$\begin{aligned} \mathbf {v} (t)&= \int _{0}^{t} \left( \mu _{{\tilde{D}}-2}^{-1} \left( \mathrm{e}^{r(t-u)} - \mathrm{e}^{-\gamma (t-u)} \right) \right) ^2 \mathrm{e}^{ \varvec{{\varOmega }} u} \mathbf {c} _{nn} \; \mathrm {d}u\nonumber \\&\quad + 2 \int _{0}^{t} \mu _{{\tilde{D}}-2}^{-1} \left( \mathrm{e}^{r(t-u)} - \mathrm{e}^{-\gamma (t-u)} \right) \mathrm{e}^{-\gamma (t-u)} \mathrm{e}^{ \varvec{{\varOmega }} u} \mathbf {c} _{1n} \; \mathrm {d}u\nonumber \\&\quad + \int _{0}^{t} \mathrm{e}^{-2 \gamma (t-u)} \mathrm{e}^{ \varvec{{\varOmega }} u} \mathbf {c} _{11} \; \mathrm {d}u . \end{aligned}$$

Now

10.18 $$\begin{aligned} \varvec{{\varOmega }} \mathbf {n} _2 = \tau \mu _{({\tilde{D}}-1)^2+1} \mathbf {n} - (2\tau + \gamma ) \mathbf {n} _2, \end{aligned}$$

so, using Proposition 1, with $\varvec{M} = \varvec{{\varOmega }} , \mathbf {x} = \mathbf {n} _2, \, \mathbf {y} = \mathbf {n} , a=-(2\tau +\gamma ), b=\tau \mu _{({\tilde{D}}-1)^2+1}$ and $c=r$, and recalling from (3.4) that $r+\gamma =\mu _{{\tilde{D}}-2}\tau$ so $a-c=-\tau (2+\mu _{{\tilde{D}}-2})=-\tau \mu _{{\tilde{D}}}$, we have

10.19 $$\begin{aligned} \mathrm{e}^{ \varvec{{\varOmega }} u} \mathbf {n} _2 = \mu _{{\tilde{D}}}^{-1}\mu _{({\tilde{D}}-1)^2+1} \left( \mathrm{e}^{ru} - \mathrm{e}^{-(2\tau +\gamma )u} \right) \mathbf {n} + \mathrm{e}^{-(2\tau +\gamma )u} \mathbf {n} _2 . \end{aligned}$$

Hence, using also (3.14),

10.20 $$\begin{aligned} \mathrm{e}^{ \varvec{{\varOmega }} u} \mathbf {c} _{11}&= \mu _{{\tilde{D}}-2}^{-1}\left( \gamma +\tau \mu _{{\tilde{D}}-2}\right) \mathrm{e}^{ru} \mathbf {n} +\gamma \mathrm{e}^{-\gamma u}\left( \mathbf {1}-\mu _{{\tilde{D}}-2}^{-1} \mathbf {n} \right) ,\nonumber \\ \mathrm{e}^{ \varvec{{\varOmega }} u} \mathbf {c} _{1n}&= \left( \gamma +\tau \mu _{{\tilde{D}}-2}\right) \mathrm{e}^{ru} \mathbf {n} ,\nonumber \\ \mathrm{e}^{ \varvec{{\varOmega }} u} \mathbf {c} _{nn}&= \left( \tau \mu _{({\tilde{D}}-2)^2}+\gamma \mu _{{\tilde{D}}}^{-1} \mu _{({\tilde{D}}-1)^2+1}\right) \mathrm{e}^{ru} \mathbf {n} +\gamma \mathrm{e}^{-(2\tau +\gamma )u}\left( \mathbf {n} _2-\mu _{{\tilde{D}}}^{-1} \mu _{({\tilde{D}}-1)^2+1} \mathbf {n} \right) . \end{aligned}$$

Let $I_1(t)=\int _0^t \mathrm{e}^{-2 \gamma (t-u)} \mathrm{e}^{ru} \; \mathrm {d}u, I_2(t)=\int _0^t \mathrm{e}^{-2 \gamma (t-u)} \mathrm{e}^{-\gamma u} \; \mathrm {d}u, I_3(t)=\int _0^t\left( \mathrm{e}^{r(t-u)}\right. \left. -\mathrm{e}^{-\gamma (t-u)}\right) \mathrm{e}^{-\gamma (t-u)} \mathrm{e}^{ru} \; \mathrm {d}u$, $I_4(t)=\int _0^t \left( \mathrm{e}^{r(t-u)}-\mathrm{e}^{-\gamma (t-u)}\right) ^2 \mathrm{e}^{ru} \; \mathrm {d}u$ and $I_5(t)=\int _0^t \left( \mathrm{e}^{r(t-u)}-\mathrm{e}^{-\gamma (t-u)}\right) ^2 \mathrm{e}^{-(2\tau +\gamma )u}\; \mathrm {d}u$. It is easily verified that these integrals are given by (4.6). Substituting (10.20) into (10.17) and using (4.6) yields (4.4).

## Appendix 4: Derivation of covariance function

For $t \ge 0$, let $\mathbf {u} (t)$ be the column vector whose *k*th element is $\mathbf {1} ^{\!\top } \varvec{V} ^{(k)}(t) \mathbf {n}$. Arguing as in the derivation of (10.17) yields

10.21 $$\begin{aligned} \mathbf {u} (t)&= \int _{0}^{t} \mu _{{\tilde{D}}-2}^{-1} \left( \mathrm{e}^{r(t-u)} - \mathrm{e}^{-\gamma (t-u)} \right) \mathrm{e}^{r(t-u)} \mathrm{e}^{ \varvec{{\varOmega }} u} \mathbf {c} _{nn} \; \mathrm {d}u\nonumber \\&\quad + \int _{0}^{t} \mathrm{e}^{-\gamma (t-u)} \mathrm{e}^{r(t-u)} \mathrm{e}^{ \varvec{{\varOmega }} u} \mathbf {c} _{1n} \; \mathrm {d}u. \end{aligned}$$

Using (10.20) now gives

10.22 $$\begin{aligned} \mathbf {u} (t)= \beta _1(t) \mathbf {n} + \beta _2(t) \mathbf {n} _2, \end{aligned}$$

where

10.23 $$\begin{aligned} \beta _1(t)&= \gamma \mu _{{\tilde{D}}-2}^{-1}\left[ \mu _{{\tilde{D}}}^{-1}\mu _{({\tilde{D}}-1)^2+1}(I_7(t)-I_8(t))+\mu _{{\tilde{D}}-2}I_6(t)\right] \nonumber \\&\quad +\tau \mu _{{\tilde{D}}-2}^{-1}\left[ \mu _{({\tilde{D}}-2)^2}I_7(t)+ \mu _{{\tilde{D}}-2}^2 I_6(t)\right] ,\nonumber \\ \beta _2(t)&= \gamma \mu _{{\tilde{D}}-2}^{-1} I_8(t), \end{aligned}$$

with $I_6(t)=\int _0^t \mathrm{e}^{- \gamma (t-u)} \mathrm{e}^{r (t-u)}\mathrm{e}^{ru} \; \mathrm {d}u, I_7(t)=\int _0^t \left( \mathrm{e}^{r(t-u)} - \mathrm{e}^{-\gamma (t-u)}\right) \mathrm{e}^{r(t-u)}\mathrm{e}^{ru} \; \mathrm {d}u$ and $I_8(t)=\int _0^t \left( \mathrm{e}^{r(t-u)} - \mathrm{e}^{-\gamma (t-u)}\right) \mathrm{e}^{r(t-u)} \mathrm{e}^{-(2\tau +\gamma )u}\; \mathrm {d}u$. It is easily verified that these integrals are given by (5.5). The expression (5.3) for the covariance follows using (5.2), (10.21), (10.22) and (4.3).

## Appendix 5: Late behaviour of subcritical survival probabilities

To bound the late probabilities of survival in the subcritical case, first note that due to the inability of type-0 individuals to transmit we have

10.24 $$\begin{aligned} q_0(t) = \mathrm{e}^{-\gamma t} . \end{aligned}$$

Recalling that $q_k(t)=1-\pi _k(t)$, it follows from (7.2), with $k_{\mathrm {max}}=\infty$, that for $k>0$,

10.25 $$\begin{aligned} \frac{\mathrm {d}q_k}{\mathrm {d}t} = -(\gamma + \tau k)q_k + \tau k q_{k-1} + \tau k (1-q_{k-1})\sum _{l=0}^{\infty } \tilde{p}_{l+1}q_l . \end{aligned}$$

This leads to the equation for $k=1$ in the form

10.26 $$\begin{aligned} \frac{\mathrm {d}q_1}{\mathrm {d}t} = r q_1 + h(t) , \end{aligned}$$

where

10.27 $$\begin{aligned} h(t) = \underbrace{\tau \mathrm{e}^{-\gamma t} \left( 1 + \tilde{p}_1 - \sum _{k=0}^{\infty } \tilde{p}_{l+1}q_k(t)\right) }_{h_1(t)} + \underbrace{\tau \sum _{k=1}^{\infty } \tilde{p}_{l+1} \left( q_k(t) - k q_1(t)\right) }_{h_2(t)}.\qquad \quad \end{aligned}$$

We assume first that $r>-\gamma$. Integrating (10.26) gives

$$\begin{aligned} \mathrm{e}^{-r t}q_1(t) = 1 - \int _{0}^{t} \mathrm{e}^{-rt} h(u)\mathrm {d}u . \end{aligned}$$

The limit $\lim _{t\rightarrow \infty }\mathrm{e}^{-rt}q_1(t)$ therefore exists if *r* is within the region of convergence of the Laplace transform of *h*. Considering $h_1(t)$ in (10.27), since $q_k(t)\in [0,1]$, for all *k*, we have that $\tilde{p}_1 \le h_1(t) \le \tau (1+\tilde{p}_1) \mathrm{e}^{-\gamma t}$, so the Laplace transform of $h_1$ converges by the assumption that $r>-\gamma$.

Now considering $h_2$, we follow Windridge (2015) and consider an initial individual with effective degree *k*, and stubs labelled by integer $i=1,2,\ldots ,k$. Let $T_i$ be the time that the individual and its progeny through stub *i* exist, and let *T* be the lifetime of the branching process. Then, using the Bonferroni inequalities as in Windridge (2015), for $k \ge 1$,

10.28 $$\begin{aligned} \{T>t\}= & {} \bigcup _{i=1}^{k} \{T_i>t\} ,\!\quad q_k(t) \le k q_1(t) \quad \! \text{ and } \ q_k(t) \!\ge \! k q_1(t) - k^2 \mathbb {P}(T_1>t, T_2>t) . \nonumber \\ \end{aligned}$$

Therefore, we have that

$$\begin{aligned} 0 \le h_2(t) \le \tau \mu _{({\tilde{D}}-1)^2} \mathbb {P}(T_1>t, T_2>t) . \end{aligned}$$

Now, from (7.3) we have that for some constant $\kappa$,

10.29 $$\begin{aligned} q_k(t) \le k \kappa \mathrm{e}^{rt} . \end{aligned}$$

Writing *R* for the lifetime of the initial infective individual we have that

$$\begin{aligned} \mathbb {P}(T_1>t, T_2>t) = \mathbb {P}(R>t) + \mathbb {P}(T_1>t, T_2>t, R\le t). \end{aligned}$$

We then recall that $\mathbb {P}(R>t) = q_0(t) = \mathrm{e}^{-\gamma t}$, after which the argument follows closely that of Windridge (2015) (as in the derivation of (10.36) below) and we find that

10.30 $$\begin{aligned} \lim _{t \rightarrow \infty } \int _0^\infty \mathrm{e}^{-rt}\mathbb {P}(T_1>t, T_2>t) \mathrm {d}t < \infty . \end{aligned}$$

Thus the Laplace transform of $h_2$ converges at *r*, whence $\lim _{t\rightarrow \infty }\mathrm{e}^{-rt}q_1(t)$ is equal to a finite constant, *c* say. Note that *c* is strictly positive since $c \ge \lim _{t \rightarrow \infty }\mathrm{e}^{-rt}{\hat{q}}_1(t)={\hat{c}}>0$. Further, (10.30) implies that $\lim _{t \rightarrow \infty }\mathrm{e}^{-rt}\mathbb {P}(T_1>t, T_2>t)=0$, and it follows from the two inequalities in (10.28) that, $\lim _{t \rightarrow \infty } \mathrm{e}^{-rt}q_k(t)=kc$, for $k \ge 0$, proving (7.5).

We consider now the case when $r<-\gamma$. For $t\ge 0$ and $k=0,1,\ldots$, write

10.31 $$\begin{aligned} q_k(t)=\mathrm{e}^{-\gamma t}+{\tilde{q}}_k(t) \qquad \text{ and } \qquad {\tilde{u}}_k(t)=\mathrm{e}^{\gamma t}{\tilde{q}}_k(t), \end{aligned}$$

so ${\tilde{q}}_k(t)$ is the probability that the branching process has survived to time *t* but the initial individual has not, and ${\tilde{u}}_0(t)=0$ for all $t \ge 0$. It follows using (10.25) that

10.32 $$\begin{aligned} \frac{\mathrm {d}{\tilde{u}}_1}{\mathrm {d}t} = \tau \left[ -{\tilde{u}}_1+(1-\mathrm{e}^{-\gamma t})\left( 1+\sum _{l=1}^{\infty } \tilde{p}_{l+1}{\tilde{u}}_l\right) \right] . \end{aligned}$$

The Bonferroni inequalities yield that, for $k \ge 1$,

10.33 $$\begin{aligned} k {\tilde{q}}_1(t)- k^2 \mathbb {P}(R<t, T_1>t, T_2>t) \le {\tilde{q}}_k(t) \le k {\tilde{q}}_1(t) , \end{aligned}$$

so

10.34 $$\begin{aligned} 0 \le {\tilde{u}}_k(t) \le k {\tilde{u}}_1(t), \end{aligned}$$

and (10.32) implies that

$$\begin{aligned} \frac{\mathrm {d}{\tilde{u}}_1}{\mathrm {d}t} \le \tau \left( 1+\mu _{{\tilde{D}}-2}{\tilde{u}}_1\right) , \end{aligned}$$

whence, for all $t \ge 0$, recalling that $\mu _{{\tilde{D}}-2}<0$,

10.35 $$\begin{aligned} 0 \le {\tilde{u}}_1(t) \le -\frac{1}{\mu _{{\tilde{D}}-2}}\left( 1-\mathrm{e}^{\tau \mu _{{\tilde{D}}-2} t}\right) \le -\frac{1}{\mu _{{\tilde{D}}-2}}. \end{aligned}$$

Conditioning on the lifetime of the initial individual in the branching process,

$$\begin{aligned} \mathbb {P}(R \le t, T_1>t, T_2>t)&=\int _{u=0}^t \gamma \mathrm{e}^{-\gamma u} \left[ \int _{v=0}^u \tau \mathrm{e}^{-\tau v}q_1(t-v)\mathrm {d}v\right] ^2 \mathrm {d}u\\&\le \int _{u=0}^t \gamma \mathrm{e}^{-\gamma u} \left[ \int _{v=0}^u \tau \mathrm{e}^{-\tau v}\left( -\frac{\mu _{{\tilde{D}}-1}}{\mu _{{\tilde{D}}-2}}\right) \mathrm{e}^{-\gamma (t-v)}\mathrm {d}v\right] ^2 \mathrm {d}u, \end{aligned}$$

since (10.31) and (10.35) imply that $q_1(t) \le -\frac{\mu _{{\tilde{D}}-1}}{\mu _{{\tilde{D}}-2}} \mathrm{e}^{-\gamma t}$. Elementary integration then shows that there exists $c'=c'(\mu , \tau ,\mu _{{\tilde{D}}})<\infty$ such that, for all $t \ge 0$,

10.36 $$\begin{aligned} \mathrm{e}^{\gamma t}\mathbb {P}(R \le t, T_1>t, T_2>t) \le {\left\{ \begin{array}{ll} c'\mathrm{e}^{-\min \{\gamma ,2\tau \}t} &{} \text { if } \gamma \ne 2 \tau ,\\ c' t \mathrm{e}^{-\gamma t} &{} \text { if } \gamma = 2 \tau . \end{array}\right. } \end{aligned}$$

The differential equation (10.32) may be written in the form

$$\begin{aligned} \frac{\mathrm {d}{\tilde{u}}_1}{\mathrm {d}t} = \tau (1+{\tilde{u}}_1)-\tau \underbrace{\mathrm{e}^{-\gamma t}\left( 1+\sum _{l=1}^{\infty } \tilde{p}_{l+1}{\tilde{u}}_l\right) }_{h_3(t)}-\tau \underbrace{\sum _{l=2}^\infty \tilde{p}_{l+1}(l {\tilde{u}}_1-{\tilde{u}}_l)}_{h_4(t)}, \end{aligned}$$

whence

10.37 $$\begin{aligned} {\tilde{u}}_1(t)=-\frac{1}{\mu _{{\tilde{D}}-2}}\left( 1-\mathrm{e}^{\tau \mu _{{\tilde{D}}-2} t}\right) -\tau \mathrm{e}^{\tau \mu _{{\tilde{D}}-2} t} \int _0^t \mathrm{e}^{-\tau \mu _{{\tilde{D}}-2} u}\left( h_3(u)+h_4(u)\right) \mathrm {d}u. \nonumber \\ \end{aligned}$$

Now (10.34) and (10.35) imply that $0 \le h_3(t) \le -\frac{1}{\mu _{{\tilde{D}}-2}}\mathrm{e}^{- \gamma t}$, whence

10.38 $$\begin{aligned} \lim _{t \rightarrow \infty } \mathrm{e}^{\tau \mu _{{\tilde{D}}-2} t}\int _0^t \mathrm{e}^{-\tau \mu _{{\tilde{D}}-2} u}h_3(u)\mathrm {d}u=0. \end{aligned}$$

Further, it follows using (10.33) and (10.36) that $0 \le h_4(t) \le \mu _{({\tilde{D}}-1)^2} c'\mathrm{e}^{-\min \{\gamma ,2\tau \}t}$, where $\mathrm{e}^{-\min \{\gamma ,2\tau \}t}$ is replaced by $t \mathrm{e}^{-\gamma t}$ if $\gamma = 2 \tau$, whence (10.38) also holds when $h_3(u)$ is replaced by $h_4(u)$. Letting $t \rightarrow \infty$ in (10.37) yields (7.6).

## Footnotes

1 There is a small error in the latter—in the expression for $b_{jk}^{(i)}$ on p. 203 of Athreya and Ney (1972), $\delta _{jk}-b_{ij}\delta _{ik}-b_{ik}\delta _{ij}+\delta _{ij}\delta _{ik}$ should be replaced by $b_{ij}\delta _{jk}-b_{ik}\delta _{ij}-b_{ij}\delta _{ik}$.

## References

[CR1] Athreya KB, Ney PE (1972). Branching processes.

[CR2] Bailey NTJ (1957). The mathematical theory of epidemics.

[CR3] Ball F, Donnelly P (1995). Strong approximations for epidemic models. Stoch Process Appl.

[CR4] Ball F, Neal P (2008). Network epidemic models with two levels of mixing. Math Biosci.

[CR5] Barbour A, Reinert G (2013). Approximating the epidemic curve. Electron J Probab.

[CR6] Black AJ, House T, Keeling MJ, Ross JV (2014). The effect of clumped population structure on the variability of spreading dynamics. J Theor Biol.

[CR7] Bohman T, Picollelli M (2012). SIR epidemics on random graphs with a fixed degree sequence. Random Struct Algorithms.

[CR8] Constable GWA, McKane AJ (2014). Fast-mode elimination in stochastic metapopulation models. Phys Rev E.

[CR9] Daley DJ, Vere-Jones D (1988). An introduction to the theory of point processes. Probability and its applications.

[CR10] Danon L, Ford AP, House T, Jewell CP, Keeling MJ, Roberts GO, Ross JV, Vernon MC (2011). Networks and the epidemiology of infectious disease. Interdiscip Perspect Infect Dis.

[CR11] Decreusefond L, Dhersin JS, Moyal P, Tran VC (2012). Large graph limit for an SIR process in random network with heterogeneous connectivity. Annal Appl Probab.

[CR12] Dorman K, Sinsheimer J, Lange K (2004). In the garden of branching processes. SIAM Rev.

[CR13] Durrett R (2007). Random graph dynamics.

[CR14] Eames KTD, Keeling MJ (2002). Modeling dynamic and network heterogeneities in the spread of sexually transmitted diseases. PNAS.

[CR15] Ethier SN, Kurtz TG (1986). Markov processes: characterization and convergence. Wiley series in probability and mathematical statistics.

[CR16] Graham M, House T (2014). Dynamics of stochastic epidemics on heterogeneous networks. J Math Biol.

[CR17] Grimmett GR, Stirzaker DR (2001). Probability and random processes.

[CR18] Heesterbeek H, Anderson RM, Andreasen V, Bansal S, De Angelis D, Dye C, Eames KTD, Edmunds WJ, Frost SDW, Funk S, Hollingsworth TD, House T, Isham V, Klepac P, Lessler J, Lloyd-Smith JO, Metcalf CJE, Mollison D, Pellis L, Pulliam JRC, Roberts MG, Viboud C, Isaac Newton Institute IID collaboration (2015) Modelling infectious disease dynamics in the complex landscape of global health. Science 347(6227):aaa4339 10.1126/science.aaa4339 PMC4445966 25766240

[CR19] Heinzmann D (2009). Extinction times in multitype Markov branching processes. J Appl Probab.

[CR20] Holme P (2013). Extinction times of epidemic outbreaks in networks. PLoS ONE.

[CR21] House T, Keeling MJ (2010). Insights from unifying modern approximations to infections on networks. J R Soc Interface.

[CR22] Janson S, Luczak M, Windridge P (2014). Law of large numbers for the SIR epidemic on a random graph with given degrees. Random Struct Algorithms.

[CR23] Klepac P, Metcalf CJE, McLean AR, Hampson K (2013) Towards the endgame and beyond: complexities and challenges for the elimination of infectious diseases. Philos Trans R Soc Lond B Biol Sci 368(1623): 20120137 10.1098/rstb.2012.0137 PMC3720036 23798686

[CR24] Lindquist J, Ma J, Driessche P, Willeboordse FH (2010). Effective degree network disease models. J Math Biol.

[CR25] Miller JC (2011). A note on a paper by Erik Volz: SIR dynamics in random networks. J Math Biol.

[CR26] Miller JC (2014). Epidemics on networks with large initial conditions or changing structure. PLoS ONE.

[CR27] Miller JC, Kiss IZ (2014). Epidemic spread in networks: existing methods and current challenges. Math Model Nat Phenom.

[CR28] Miller JC, Slim AC, Volz EM (2012). Edge-based compartmental modelling for infectious disease spread. J R Soc Interface.

[CR29] Molloy M, Reed B (1995). A critical point for random graphs with a given degree sequence. Random Struct Algorithms.

[CR30] Murray JD (1984). Asymptotic analysis, applied mathematical sciences.

[CR31] Murray JD (2002). Mathematical biology I.

[CR32] Murray JD (2003). Mathematical biology II.

[CR33] Newman MEJ (2002). Assortative mixing in networks. Phys Rev Lett.

[CR34] Newman MEJ (2002). Spread of epidemic disease on networks. Phys Rev E.

[CR35] O’Neill PD, Roberts GO (1999). Bayesian inference for partially observed stochastic epidemics. J R Stat Soc A.

[CR36] Parsons TL, Rogers T (2015) Dimension reduction via timescale separation in stochastic dynamical systems. [arXiv:1510.07031]

[CR37] Ross JV, Taimre T, Pollett PK (2006). On parameter estimation in population models. Theor Popul Biol.

[CR38] Volz EM (2008). SIR dynamics in random networks with heterogeneous connectivity. J Math Biol.

[CR39] Waugh WAO (1958). Conditioned Markov processes. Biometrika.

[CR40] Windridge P (2015) The extinction time of a subcritical branching process related to the SIR epidemic on a random graph. J Appl Probab 52(4):1195–1201

